# Reinforcement of Gametic Isolation in *Drosophila*

**DOI:** 10.1371/journal.pbio.1000341

**Published:** 2010-03-23

**Authors:** Daniel R. Matute

**Affiliations:** Department of Ecology and Evolution, The University of Chicago, Chicago, Illinois, United States of America; University of Edinburgh, United Kingdom

## Abstract

Reinforcement, a process by which natural selection increases reproductive isolation between populations, has been suggested to be an important force in the formation of new species. However, all existing cases of reinforcement involve an increase in mate discrimination between species. Here, I report the first case of reinforcement of postmating prezygotic isolation (i.e., barriers that act after mating but before fertilization) in animals. On the slopes of the African island of São Tomé, *Drosophila yakuba* and its endemic sister species *D. santomea* hybridize within a well-demarcated hybrid zone. I find that *D. yakuba* females from within this zone, but not from outside it, show an increase in gametic isolation from males of *D. santomea*, an apparent result of natural selection acting to reduce maladaptive hybridization between species. To determine whether such a barrier could evolve under laboratory conditions, I exposed *D. yakuba* lines derived from allopatric populations to experimental sympatry with *D. santomea*, and found that both behavioral and gametic isolation become stronger after only four generations. Reinforcement thus appears to be the best explanation for the heightened gametic isolation seen in sympatry. This appears to be the first example in animals in which natural selection has promoted the evolution of stronger interspecific genetic barriers that act after mating but before fertilization. This suggests that many other genetic barriers between species have been increased by natural selection but have been overlooked because they are difficult to study.

## Abstract 2

*D. santomea* and *D. yakuba* provide an example of reinforcement for a postmating-prezygotic trait in an organism that has internal fertilization. This work shows that reinforcement of barriers other than sexual and other forms of premating isolation is possible.

## Author Summary

What stops newly formed species from interbreeding? Answering this question is fundamental to our understanding of speciation. One mechanism is that where such would-be species meet, the barriers against interbreeding are reinforced by natural selection (e.g., leading to greater mate discrimination). On the slopes of the African island of São Tomé, *Drosophila yakuba* and its sister species *D. santomea* hybridize within a well-demarcated hybrid zone. I found that *D. yakuba* females from within this zone, but not from outside it, show an increase in gametic isolation from males of *D. santomea*, such that before fertilization, the females deplete sperm from *D. santomea* males faster than from conspecific males. Consequently, there are fewer progeny produced from interspecific matings. By experimentally evolving the populations, I also show that such postmating isolation can rapidly evolve. Natural selection, therefore, has promoted the evolution of stronger interspecific genetic barriers that act after mating but before fertilization. *D. santomea* and *D. yakuba*, then, appear to represent an example of reinforcement for a postmating-prezygotic trait in an organism that has internal fertilization. This work shows that reinforcement of barriers other than sexual and other forms of premating isolation is possible. This also suggests that there are many “cryptic” barriers to gene flow that might be increased by natural selection in areas where species overlap and hybridize.

## Introduction

The evolutionary process of “reinforcement,” often suggested as an important component of speciation, involves the strengthening by natural selection of prezygotic isolation between closely related taxa in response to maladaptive hybridization [1]–[4]. Reinforcement has often been inferred from a pattern of “reproductive character displacement,” in which individuals of different species are more behaviorally isolated if they come from the area where two species overlap (sympatric) than from areas outside each other's range (allopatric; [1]–[8]). Reinforcing selection, however, need not be limited to increasing premating isolation: other reproductive barriers that act after mating, such as gametic isolation, can also be reinforced [9]–[14]. Lorch and Servedio [15], for example, proposed that a species preference for fertilizing the gametes of conspecific versus heterospecific individuals could evolve through a reinforcement-like process, depending on the nature of selection against heterospecific matings. Here, I report the first, to my knowledge, apparent case of reinforcement in the wild of a genetic barrier—reduced production of hybrid eggs—that acts after mating but before fertilization; and I also demonstrate that the evolution of this form of gametic isolation can occur in the laboratory.

I looked for evidence of reinforcement in postmating-prezygotic isolating mechanisms in two African species of *Drosophila* in the *melanogaster* subgroup: *D. yakuba* and *D. santomea*. *D. yakuba* is widespread throughout sub-Saharan Africa and has extended its range to neighboring islands, including the Gulf of Guinea islands in the eastern Atlantic Ocean [16]. *D. santomea*, the closest relative of *D. yakuba*, is endemic to São Tomé, a small (860 km^2^) volcanic island 255 km west of Gabón. Molecular data show that *D. yakuba* and *D. santomea* diverged about 400,000 y ago [17],[18]. On the extinct volcano of Pico de São Tomé, *D. yakuba* occurs at elevations below 1,450 m, and is also common in the lowlands, villages, and plantations. In contrast, *D. santomea* occupies the mist forests at elevations between 1,153 and 1,800 m [16]–[19]. These species are unique within *Drosophila* in showing a well-demarcated hybrid zone.

Previous studies uncovered at least 11 distinct reproductive barriers that act over the entire life cycle, ranging from habitat isolation to hybrid dysfunction, although no single barrier completely impedes gene flow [20]–[24]. Five known barriers are of the postmating-prezygotic form [22],[24], including both competitive (conspecific sperm precedence [CSP]) and noncompetitive mechanisms (lower production of eggs after heterospecific matings). The *yakuba–santomea* species pair is ideal for studying reinforcement because it meets the requirements that 1) mating and introgression occur between the species in nature (as observed in the hybrid zone between *yakuba* and *santomea*) and 2) that hybridization be costly (all male hybrids are sterile). Previous studies of these species have failed to find evidence of reinforcement in premating barriers [20], but there was no search for reinforcement in postmating-prezygotic barriers.

Here, I report that reinforcement for a form of postmating-prezygotic isolation—gametic isolation—has apparently evolved in natural populations of *D. yakuba* sympatric with the sister species *D. santomea*. I demonstrate a clear fitness advantage for those individuals who have increased gametic isolation, and this advantage apparently leads to a remarkably rapid evolution of gametic isolation in laboratory populations. This appears to be the first known example in animals of the evolutionary increase of interspecific genetic barriers that act after mating but before fertilization.

## Materials and Methods

### Gametic Isolation of Naturally Collected Lines

**Figure 1:**
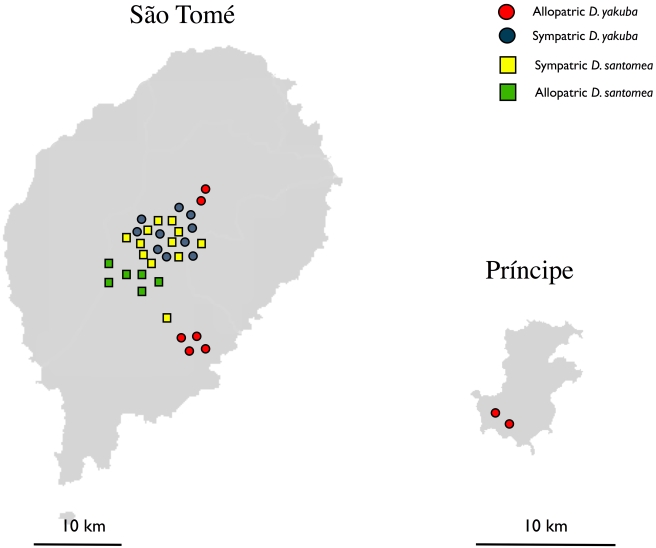
Map depicting the *D. yakuba* and *D. santomea* collection sites in the São Tomé and Príncipe islands. Lines collected in the African continent are not shown. Additional information about the collection sites can be found in Table S1.

Figure 1 and Table S1 describe the collection sites of the stocks used in this study. I used isofemale lines to study the among-line component of variation in gametic isolation. (The among-population variation was not evaluated because it is not possible to sample multiple populations from the area of sympatry.) Females of each line from each species were mated to both conspecific and heterospecific males to estimate egg number produced by each type of cross. I collected virgin males and females under CO_2_ anesthesia and kept them for 3 d in single-sex groups of 20 flies. On day 4, I aspirated flies into fresh food-containing vials, with one female and one male per vial. All copulations were watched to ensure that they were not abnormally short. To prevent females from remating, males were removed from a vial by aspiration after mating. After 1 h, I ended the observations and discarded females who did not mate. Each mated female was allowed to oviposit for 24 h in a vial, after which I counted the total number of eggs laid and transferred the female to a fresh vial. The counting was repeated daily for 10 d. In interspecific crosses, the reduced number of offspring constitutes a noncompetitive form of gametic reproductive isolation, as each female carries sperm from only one male [22],[24]. Twelve females were scored for each cross.

### Rate of Sperm Depletion (or Death)

One way to measure the efficiency of sperm storage or survival is to estimate the proportion of eggs laid every day that hatch, following the decline in this statistic over time [24]. To this end, I used six *D. yakuba* lines (three allopatric and three sympatric) and three *D. santomea* lines, measuring the decline of egg hatchability for all the possible *D. yakuba* × *D. santomea* crosses. For each cross, I produced 100 inseminated females, divided into five subgroups of 20 females. Each subgroup was transferred without anesthesia to colored medium. Eggs were collected every 24 h, and the hatchability of each batch was measured daily for 10 d.

Heterogeneity in hatchability among crosses was analyzed by fitting a minimal random linear mixed model (LMM) [25] to the hatchability of eggs laid each day. I analyzed five main effects—geographic origin of female (sympatric vs. allopatric populations), geographic origin of male, female line nested within geographic origin, male line nested within geographic origin, and days after mating—as well as all interactions between these factors. The effect due to differences between groups of females was considered random. I analyzed the data following the maximum-likelihood model simplification approach of Crawley [26],[27], in which the full model containing all factors and interactions was fitted and then simplified by a series of stepwise deletions, starting with the highest-order interaction and progressing to lower-order interaction terms and then to main effects. The critical probabilities for retaining factors and determining whether effects or interactions were significant were 5% for main effects, 1% for two-way interactions, and 0.5% for three-way interactions [28]. To assess whether slopes (i.e., the rate at which hatchability decays along time) differ between matings, I formulated two models that differed in the assumptions about these slopes and compared the models using a likelihood ratio test (LRT). Model 1 was a full factorial analysis (i.e., different slopes and intercepts for each of the possible crosses), whereas Model 2 assumed different intercepts but identical slopes (rate of decline of fecundity). Finally, to determine whether different treatments produced differences in initial hatchability (as a proxy for the amount of sperm transferred during heterospecific matings), I analyzed hatchability data from the first day using a one-way ANOVA, with hatchability as the response and two fixed effects (origin of the female, and female line nested within origin).

### Selective Advantage

To study whether an initial interspecific mating had any effects on the fertility of *D. yakuba* females after a second conspecific mating, I scored the egg production of heterospecifically mated females. After 4 d, interspecifically mated females were remated to *D. yakuba* males (from the same line than the female), and I scored the number of eggs laid during the subsequent 10 d. Eggs were counted every 24 h over the entire 14-d period. This analysis used 12 lines (six allopatric and six sympatric), with 25 individuals scored per line.

I analyzed differences in overall fecundity between the different crosses by fitting a nested ANOVA to the total number of eggs laid per female (the sum of heterospecific and conspecific eggs), with geographic origin of the female and female line (nested within origin) as fixed effects and variation among females within line as a random effect. To determine whether the proportion of conspecific eggs (relative to the total number of eggs) differed between sympatric and allopatric lines, I followed the same procedure used to analyze total fertility, but fitted the model to the number of conspecific eggs laid (i.e., eggs laid after the second mating).

### Experimental Sympatry

To test whether natural selection on gametic isolation could have been responsible for the observed reproductive character displacement in natural populations, I kept seven populations of *D. yakuba*, originally derived from allopatric populations (Table S2), in experimental sympatry with *D. santomea* for ten generations, following the design of Koopman [29] and Higgie et al. [30]. These conditions were created by maintaining four bottles per population, with each bottle containing 50 *D. yakuba* females, 50 *D. yakuba* males, 50 *D. santomea* females, and 50 *D. santomea* males. Since *D. yakuba* always outcompetes *D. santomea* under these conditions [24], I added *D. santomea* females and males to the experimental sympatry bottles each generation to maintain a constant ratio of the two species. To set up each successive generation, I collected 50 flies of each sex of *D. yakuba* (easily identifiable by pigmentation) as virgins from the experimental bottles and transferred them to a new bottle. To reconstitute the sympatry conditions, 50 *D. santomea* flies of each sex (collected as virgins from stock bottles) were added to the bottle. This procedure was followed for ten generations. Control populations of *D. yakuba* were maintained for each population (four replicates) at the same density (100 flies per bottle) but without adding *D. santomea*. The maintenance conditions and population size of *D. yakuba* were the same between experimental sympatry bottles and control groups. The strength of sexual and gametic isolation was measured every two generations using methods described previously [22],[31],[32].

Finally, I set up an internal control to make sure that elimination of hybrids was complete, i.e., there was no gene flow between the two species in the experimental bottles. Taking into account the complete sterility of F_1_ hybrid males, who lack motile sperm [17],[21], I collected *D. yakuba* females from each experimental sympatry bottle and mated them to *D. santomea* males to produce 100 F_1_ heterospecific males (♀ *D. yakuba* × ♂ *D. santomea*) every other generation. These F_1_ males were scored for sperm motility. The idea behind this test was that if motile sperm were seen, it meant that there had been gene flow between species (i.e., not all the hybrids were killed when setting up a new generation), and the bottle was discarded.

The results from this experiment were analyzed using a paired *t*-test to compare the values of gametic and sexual isolation (transformed with arcsine) between experimental populations that were exposed to *D. santomea* and the unexposed control populations.

## Results

### Gametic Isolation Is Stronger in Sympatric Than in Allopatric *D. yakuba* Females

**Figure 2:**
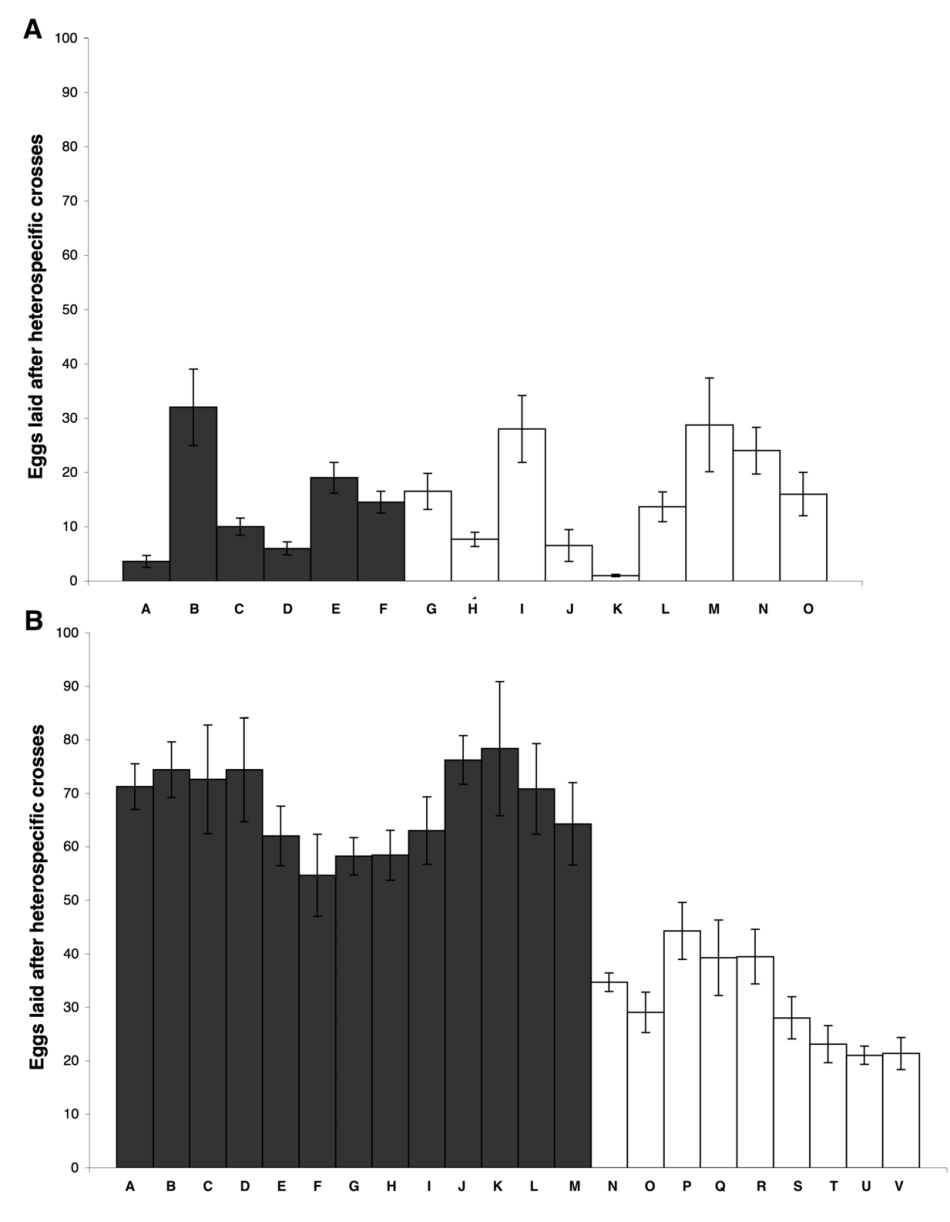
Noncompetitive gametic isolation between *D. yakuba* and *D. santomea*. (A) *D. santomea* sympatric females produced the same amount of progeny as allopatric lines after being mated to *D. yakuba* males. Bars A–F (dark grey): allopatric lines; bars G–O (white bars): sympatric lines. (B) Reproductive character displacement in *D. yakuba*. Females derived from flies sympatric to *D. santomea* yield fewer progeny than do allopatric females after being mated with *D. santomea*, suggesting a higher level of gametic isolation in sympatric females. Bars A–M (dark grey): allopatric lines; bars N–V (white): sympatric lines. Each bar represents the mean (SE) number of eggs from independent heterospecific single matings of *D. yakuba* and *D. santomea* females. The list of crosses can be found in Tables S2 and S3.

The test for reinforcement of gametic isolation involved mating *D. yakuba* and *D. santomea* females from either sympatric or allopatric populations to males of the other species and scoring the number of eggs produced by a single female—an index of the strength of noncompetitive gametic isolation—during the first 10 d after each mating (Figure 2). In *D. santomea*, I detected no heterogeneity in egg production when females were mated to *D. yakuba* males (gametic isolation) among lines (LMM, *F*_1,13_ = 1.644, *p* = 0.222, Figure 2A). In contrast, *D. yakuba* females from sympatric lines yield significantly fewer progeny than those from allopatric females when both were mated to *D. santomea* males, even when allopatric females were derived from populations close to the hybrid zone on São Tomé. *D. yakuba* females, therefore, show the pattern predicted by reinforcement of gametic isolation (LMM, *F*_1,20_ = 42.56, *p*<0.0001, Figure 2B). This suggests that in *D. yakuba*, increased gametic isolation has evolved as a response to the sympatric presence of the sister species. The results with synthetic lines (genetically heterogeneous strains of each species created by combining virgin males and females from several isofemale lines from the same location) were similar (unpublished data).

To determine whether the reduced number of hybrid eggs laid by *D. yakuba* females from sympatric populations was caused by a female trait, a male trait or the interaction of both, I randomly selected six *D. yakuba* lines (three allopatric and three sympatric) and six *D. santomea* lines, mated the *D. yakuba* females to *D. santomea* males in all the possible combinations, and performed the egg-counting protocol described above. The data were analyzed with a LMM with four fixed effects: female origin, female line (nested within female origin), and male line (nested within male origin), as well as all interactions between these factors. The minimal linear model for this design showed that there is a high degree of heterogeneity (*F*_35,396_ = 18.08, *p*<10–15) in the number of eggs produced. The results indicate that the among-line heterogeneity is explained by origin of the female (whether a population was allopatric or sympatric to *D. santomea* in the field, LMM, *F*_1,4_ = 124.818, *p* = 0.0004). The male origin effect was not significant, suggesting that the genotype of the male does not have an effect on female fertility (*F*_1,4_ = 1.822, *p* = 0.2484). More important, the interaction between female origin and male origin was not significant (*F*_16,396_ = 1.86, *p* = 0.023), demonstrating that the heterogeneity in fecundity (and therefore, the observed reproductive character displacement) is a characteristic that depends primarily on the genotype of the female, regardless of the genotype of the *D. santomea* male involved in the heterospecific cross. This kind of reinforcement is expected to be due to changes in females, because they suffer more than do males from interspecific mating [2].

### Sperm Depletion Rate

**Figure 3:**
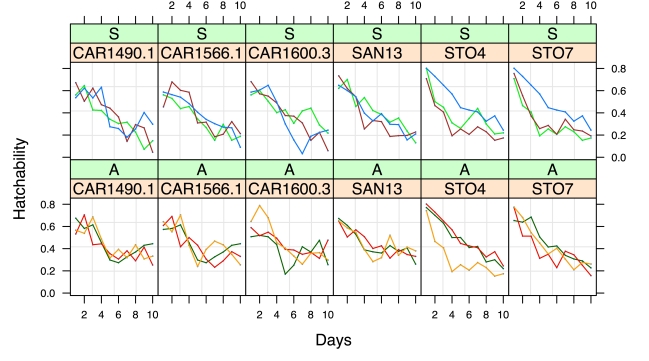
Sperm retention in allopatric and sympatric females of *D. yakuba*. A LMM was used to test for differences in sperm depletion/death over time between allopatric (A panels) and sympatric (S panels) lines of *D. yakuba*. The *D. yakuba* lines used in this experiment were SJ2 (brown), Cameroon 115 (blue), Anton 2 Principe (red), SA3 (light green), OBAT1200.15 (orange), and BAR1000.2 (dark green). I used six different *D. santomea* lines (tags highlighted in yellow) to make sure that the observed patterns were not line specific. Heterogeneity in slopes was detected between populations and was determined to be higher (i.e., faster sperm depletion/death) in sympatric than in allopatric lines.

I estimated how long a female could retain and use viable sperm when she was mated to a heterospecific versus a conspecific male. The aim of this test was to determine whether the rate at which a *D. yakuba* female lost *D. santomea* sperm—either by depletion or sperm death—differed between allopatric and sympatric *D. yakuba* lines. Figure 3 shows that heterospecific sperm loss (the most likely cause of noncompetitive gametic isolation) is more pronounced in sympatric than in allopatric lines. This conclusion rests on two results of this analysis. First, the initial hatchability of eggs did not differ between allopatric and sympatric lines (LMM; female origin: F_1,4_ = 0.585, *p* = 0.4869). There was no heterogeneity between the intercepts of these crosses, suggesting no substantive difference in number of sperm transferred. Moreover, the decline in egg hatchability over time (slope) was significantly heterogeneous (Model 1 vs. Model 2: LRT = 12.086, *p* = 5×10^−4^). This shows that interspecific sperm stored after crosses involving sympatric lines was either retained for a shorter time or became inviable more quickly than in crosses involving allopatric lines. The more rapid loss (or death) of heterospecific sperm in sympatric females is consistent with the observation that sympatric females produce fewer progeny after heterospecific crosses compared to allopatric females. Apart from noncompetitive gametic isolation, no other reproductive barrier shows the signature of reinforcement (Figures S1 and S2, Tables S4, S5, S6, and S7).

### Selective Advantage

Although the evolution of behavioral isolation is clearly advantageous in a hybrid zone when hybrids are semisterile or partly inviable, the benefits of increasing postmating-prezygotic isolation are not so obvious [2],[12],[13]. One possibility is that eliminating heterospecific sperm more quickly allows a female to remate with males of her own species, increasing her chances of passing her genes to the next generation. In such a case, alleles fostering quicker elimination of heterospecific sperm could be selectively advantageous. To check this possibility, I measured the reproductive capacity of *D. yakuba* females from both allopatric and sympatric populations that had been initially mated to heterospecific males. Four days after this first mating, these females were remated to conspecific males, and I counted the number of eggs produced every day for the next 10 d.

**Figure 4:**
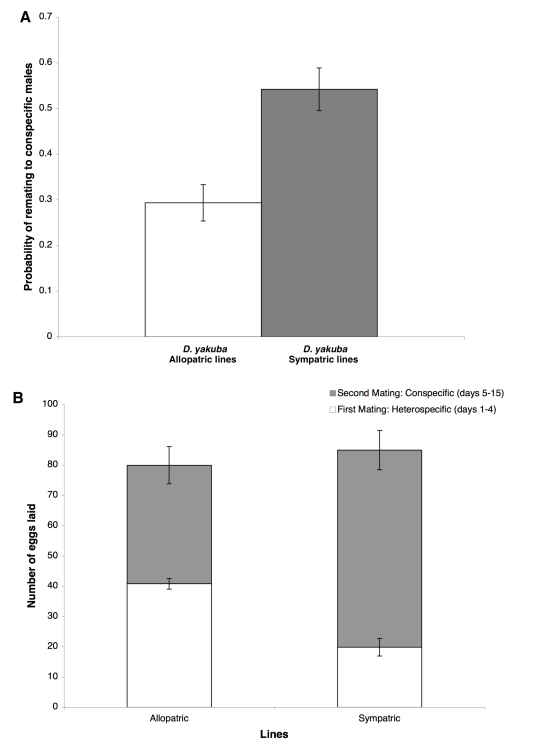
Selective advantages of enhanced gametic isolation. (A) Propensity of *D. yakuba* females to remate with a conspecific in a second, no-choice mating 4 d after an initial mating to a heterospecific male. Grey: sympatric females; white: allopatric females. (B) Mean (SE) number of eggs per *D. yakuba* female (from either sympatric or allopatric lines) sired by first (heterospecific, white) and second (conspecific, grey) male.

Data from this experiment give two kinds of support for the idea that natural selection might have increased the gametic isolation of sympatric *D. yakuba* females in nature. First, *D. yakuba* females from sympatric populations remated more quickly to conspecific males than did sympatric females (LMM on arcsine of the remating probability, *F*_1,12_ = 13.4295, *p* = 0.0032; Figure 4A). Given the CSP that acts in *D. yakuba* (in double conspecific/heterospecific matings, regardless of mating order, conspecific sperm are used in fertilization much more often than heterospecific sperm, [22]), this faster mating would markedly reduce the proportion of hybrid progeny produced, decreasing the cost of maladaptive hybridization. Second, *D. yakuba* sympatric females mated to a conspecific male for a second time produced more conspecific progeny than did allopatric females (Figure 4B). Since the total number of eggs produced did not differ between allopatric and sympatric *D. yakuba* females after two matings (LMM, *F*_1,8_ = 0.0031, *p* = 0.957), the stronger gametic isolation of sympatric females reduces the production of hybrid progeny and increases the number of (more fit) conspecific progeny that they can produce (LMM on number of eggs laid after conspecific mating: *F*_1,8_ = 9.726, *p* = 0.0143). Taken together, these results show that increased gametic isolation can provide a fitness advantage to *D. yakuba* females who are sympatric with *D. santomea*.

### Experimental Sympatry

To establish whether natural selection would increase gametic isolation in the laboratory when species were given the opportunity to hybridize, I exposed seven distinct allopatric lines of *D. yakuba* (each collected in different years and geographic localities) to experimental sympatry with *D. santomea* for ten generations. If maladaptive hybridization promotes the evolution of postmating-prezygotic isolation, and there is genetic variance for the character, we might be able to observe such isolation evolving in the experimental sympatry lines. It is important to note, however, that in this study, hybrids are rendered completely inviable, whereas a few viable hybrids have been found in the wild. Although these species do mate in the wild, female hybrids have never been found, male hybrids are completely sterile, and most hybrids have been F_1_ individuals, with only 4% of them being from backcrosses [19].

**Figure 5:**
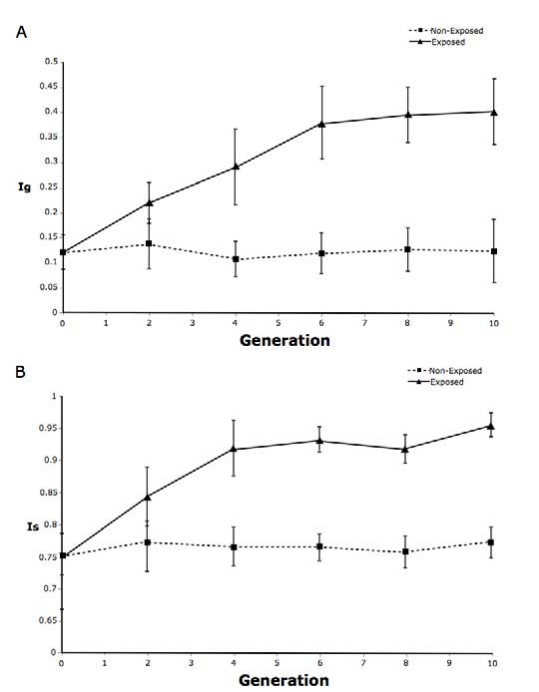
Effects of experimental sympatry on sexual (A) and gametic (B) isolation in *D. yakuba*. The strength of gametic and sexual isolation was calculated according to the indexes proposed by Chang (*I*_g_; [22]), and Coyne and Orr (*I*_s_; [32]), respectively. Means and standard errors reflect the average of the seven lines (four replicates per line). *D. yakuba* lines, which in nature are allopatric to *D. santomea*, showed significant reproductive character displacement (triangles) when exposed to *D. santomea*, whereas unexposed lines (squares) experienced no change in their degree of isolation.

*D. yakuba* females exposed to experimental sympatry evolved substantial gametic isolation within four generations, whereas unexposed *D. yakuba* populations showed no change in isolation over time (Figure 5A). This difference was highly significant (paired *t*-test: *t*_5_ = 4.32, *p* = 0.0076). I also observed a substantial increase in sexual isolation between *D. yakuba* females and *D. santomea* males in sympatric, but not in the unexposed control populations (paired *t*-test: *t*_5_ = 4.85, *p* = 0.0047; Figure 5B). This is surprising in view of the lack of evidence for reinforcement of sexual isolation of these species in nature [20]. None of the other isolating barriers examined (copulation latency and duration) changed over time (Figures S3 and S4).

Although the experimental-sympatry study demonstrates the evolution of reproductive character displacement rather than reinforcement per se, for several reasons, these results increase the likelihood that increased gametic isolation in sympatry did result from reinforcement: i) gametic isolation is a heritable trait and responds to selection, ii) increased gametic isolation similar to that seen in nature is caused by the presence of *D. santomea*, and iii) the genetic variability required for sexual and gametic isolation to evolve is present in allopatric populations. Additionally, since *D. santomea* were added to the experimental sympatry bottles each generation, I did not examine the possibility of reinforcement of gametic (or sexual isolation) in that species.

### Tests of Alternative Explanations

Increased reproductive isolation in sympatry can be generated through a variety of processes. Although reinforcement is the most commonly invoked explanation for reproductive character displacement, other processes—such as ecological character displacement and differential extinction or differential fusion— can generate the same pattern [2],[33]–[36]. Two results, however, suggest that ecological character displacement is an unlikely explanation for the observed pattern. First, to control for this possibility, I included several allopatric lines of *D. yakuba* collected from higher elevations off of São Tomé (e.g., Mount Cameroon, Pico Basile, and Nairobi, Table S1), which thus lived at elevations similar to the *D. yakuba* lines derived from the hybrid zone (Table S1). The aim of this test was to examine the possibility that the observed reproductive character displacement was a byproduct of adaptation to high elevation alone (allopatric lines collected at high elevations are represented by bars K–M, Figure 2B). These allopatric, high-elevation lines of *D. yakuba* did not, however, show elevated gametic isolation. Moreover, the results from the experimental sympatry experiment show that reproductive character displacement occurs if *D. yakuba* is exposed to *D. santomea* and when there is strong selection against the hybrids, even when the “ecology” is that of a food-filled milk bottle in the laboratory.

**Figure 6:**
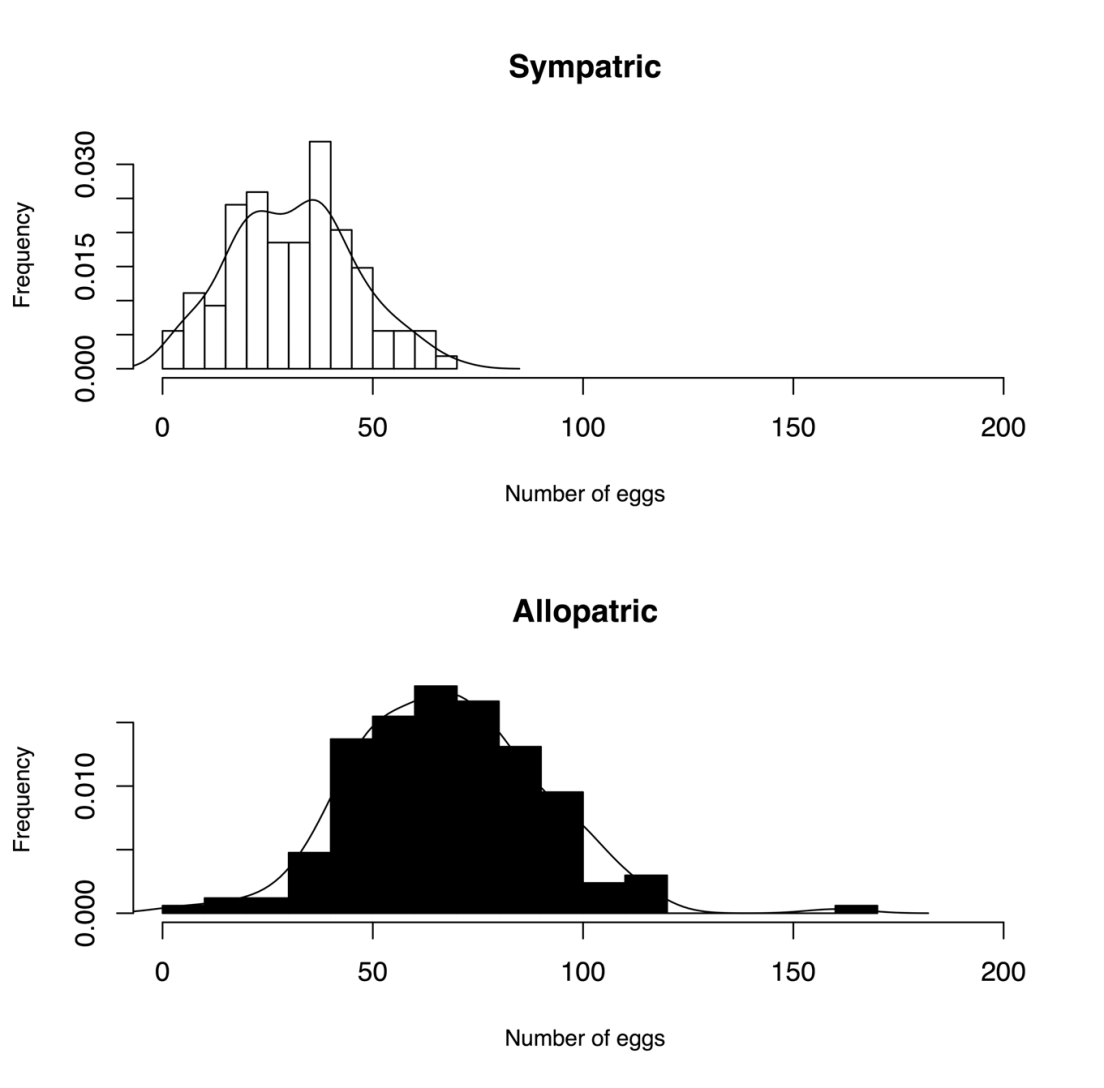
Frequency distribution of individual levels of gametic isolation levels in *D. yakuba*. The data correspond to the data shown in Figure 1 when pooled according to whether the line of each *D. yakuba* female was sympatric or allopatric.

The second possibility is that the observed range of gametic isolation reflects the results of a deme-sorting process involving differential extinction (or differential fusion) of populations based upon levels of reproductive isolation. Under this scenario, only those populations that have a high, pre-existing level of reproductive isolation will be able to colonize and persist in a region where a potentially interbreeding sister species is present. This hypothesis yields a clear prediction: if biased extinction is the driving force behind the observed differences in levels of reproductive isolation, then non-allopatric populations should show a distribution of reproductive-isolation values lying within the range of phenotypic values seen in allopatric populations [31],[32]. The data suggest that this is not a likely explanation for the elevated gametic isolation seen in sympatric *D. yakuba* lines: values of gametic isolation in sympatric lines are not a subset of that of the distribution of values of allopatric individuals (Figure 6, Figure S5, ANOVA on pooled individual values with resampling of cells: *F*_1,310_ = 341.93, *p*<1×10^−4^).

Two further lines of evidence render differential fusion/extinction an unlikely explanation. First, differential fusion predicts that premating, postmating-prezygotic, and postzygotic isolation would be stronger in sympatry than allopatry [2],[31]. Tables S4, S5, S6, and S7 show that it is not the case: the only reproductive isolating barrier appears to be strengthened in sympatry is gametic isolation. Second, to explain the existence of substantial differences in gametic isolation before secondary contact, differential fusion/deme selection would require very low levels of gene flow between populations [2],[34]. Previous studies have shown that this is not the case for *D. yakuba*, which exhibits very little population structure [17].

All these considerations render alternative possibilities, such as ecological character displacement and differential extinction/fusion, unlikely. I suggest that reinforcement is the most likely cause of the reproductive character displacement observed in *D. yakuba* populations that are sympatric to *D. santomea*.

## Discussion

Four conditions must be met before one can conclude that reinforcement is the cause of a pattern of reproductive character displacement between two species [33],[34]. First, gene flow, either current or recent, has to occur between them. Second, there must be, or have been, natural selection against maladaptive hybridization. Third, the trait causing reproductive isolation must be heritable and capable of responding to selection. Finally, one must rule out alternative explanations such as ecological character displacement. The work described above fulfills these requirements, suggesting that reinforcement for postmating prezygotic isolation has indeed evolved in populations of *D. yakuba* that are sympatric with *D. santomea*.

Some cases of reproductive character displacement of gametic isolation have been reported previously. Geyer and Palumbi [37] describe reproductive character displacement in the sequence of proteins involved in gametic interactions in sympatric populations of the sea urchin *Echinometra oblonga*. A similar example occurs in abalone and mussel species, which show a strong signature of positive selection in proteins involved in sperm–egg interaction, especially in sympatric species [38]–[42]. In all these cases, selection for local gamete coevolution (as a result of interactions between sympatric species) seems to be the driving force of speciation; however, the authors do not describe higher gametic isolation between sympatric than between allopatric *populations* of the same species pair, so it is possible that these patterns reflect processes other than reinforcement (e.g., differential fusion; [2],[34]).

*D. santomea* and *D. yakuba*, then, appear to represent the first example of reinforcement for a postmating-prezygotic trait in an organism that has internal fertilization. In this particular case, reinforcement operates when several reproductive barriers are already strong. Also, the major selection pressure seems to be direct—on the number of offspring produced—rather than indirect—on the fitness of hybrid offspring. The reason why only gametic isolation, and not sexual isolation, is reinforced in natural populations of *D. yakuba* remains an unanswered question, especially given that behavioral isolation mechanisms occurring earlier in the life history can more effectively reduce the costs of hybridization [2],[43],[44]. There are two explanations for why *D. yakuba* females show reinforced gametic isolation but no reinforced behavioral isolation. CSP reduces the cost of heterospecific matings for females, and thus reduces the likelihood reinforcement of sexual isolation [43]. It is possible that CSP reduces the likelihood of reinforcement of behavioral but not of gametic isolation; however, this seems unlikely given that CSP reduces the costs of hybridization as a whole, and its effects should reduce the likelihood of reinforcement of all mechanisms of reproductive isolation. A second possibility is that if behavioral isolation is not an effective isolating mechanism in nature, then gametic isolation can play a very prominent role on reproductive isolation, as occurs in free-spawning marine invertebrates. Again, previous inventories of reproductive isolation between *D. yakuba* and *D. santomea* and the low frequency of hybrids in nature (*I*_psi_ = 0.54 for no choice experiments; [20],[21],[23],[45]) render this explanation as unlikely.

For sexual isolation, it has been predicted that reinforcement should be stronger in the rarer species, as rarity increases the probability of mating with the wrong species [2]–[7] and thus selection to avoid maladaptive hybridization stronger. Previous studies have demonstrated that in the hybrid zone *D. yakuba* is indeed rarer than *D. santomea* [19]. Although the reproductive mechanism that is reinforced in this case is not sexual but gametic isolation, our results do comply with this prediction.

Finally, I show that gametic isolation (and not only sexual isolation) can evolve under laboratory conditions—and can do so very quickly if natural selection is strong. These results, together with some previous examples [29],[30] in which artificial sympatry promoted the evolution of reproductive character displacement, demonstrate that prezygotic isolation (both premating and postmating-prezygotic) can evolve quickly given the strong selection regime and the presence of genetic variation. Whether reinforcement would evolve if gene flow was permitted and the selection regime was weaker is an unanswered question that I am currently investigating.

To date, the study of postmating-prezygotic barriers in speciation has focused largely on documenting their existence. The processes and mechanisms that generate such reproductive mechanisms are, understandably, less well understood than those that generate premating isolation [13],[46],[47]. Previous studies have shown that postmating-prezygotic characters can evolve rapidly and that such evolution can be the result of differences in the coevolutionary trajectory between males and females among populations or species [48],[49]. Postmating-prezygotic isolation can also evolve as a byproduct of ecological divergence and be heavily influenced by the ecology of a species [36],[48]–[51].

This work shows that reinforcement of barriers other than sexual and other forms of premating isolation is possible. This suggests that there are many “cryptic” barriers to gene flow that might be increased by natural selection in areas where species overlap and hybridize.

## Supporting Information

Figure S1 **Offspring production from double matings by** ***D. yakuba*** **females I.** Mean (SE) number of offspring per *D. yakuba* female (from either sympatric or allopatric populations) sired by first (*D. yakuba*, red) and second (*D. santomea* STO.4, blue) male. The number of offspring produced during the first 4 d was subtracted from the total amount of produced progeny. The data were analyzed with a nested ANOVA in which the asin (progeny produced after the second mating/total progeny) was the response and line was nested within origin of the *D. yakuba* line (allopatric or sympatric). The results (Female origin: *F*_1,54_ = 0.069, *p* = 0.794; Female line: *F*_4,54_ = 1.188, *p* = 0.068) show no difference in the strength of CSP between sympatric and allopatric lines when *D. yakuba* is the first male.
(0.02 MB PDF)

Figure S2 **Offspring production from double matings by** ***D. yakuba*** **females II.** Mean (SE) number of offspring per *D. yakuba* female (from either sympatric or allopatric populations) sired by first (*D. santomea* STO.4, red) and second (*D. yakuba*, blue) male. The number of offspring produced during the first 4 d was subtracted from the total amount of produced progeny. The data were analyzed with a nested ANOVA in which the asin (progeny produced after the second mating/total progeny) was the response and line was nested within origin of the *D. yakuba* line (allopatric or sympatric). The results (Female origin: *F*_1,54_ = 0.643; Female line: *F*_4,54_ = 1.188, *p* = 0.327) show no difference in the strength of CSP between sympatric and allopatric lines when *D. santomea* is the first male.
(0.02 MB PDF)

Figure S3 **Effects of experimental sympatry on copulation latency in** ***D. yakuba*** **.** Means and standard errors are based on the average of the seven lines (four replicates per line).
(0.02 MB PDF)

Figure S4 **Effects of experimental sympatry on copulation duration in** ***D. yakuba*** **.** Means and standard errors are based on the average of the seven lines (four replicates per line).
(0.02 MB PDF)

Figure S5 **Frequency distributions of gametic isolation levels per** ***D. yakuba*** **line.** The title of each graph shows the lines involved in the cross (♀ *D. yakuba* × ♂ *D. santomea*). Black distributions: allopatric lines; white distributions: sympatric lines. The data shown in this figure are the same data shown in Figure 1.
(0.04 MB PDF)

Table S1 **Isofemale lines of** ***D. santomea*** **and** ***D. yakuba*** **analyzed in this study.**
(0.10 MB RTF)

Table S2 **Allopatric and sympatric crosses involving** ***D. yakuba*** **females.** Cross corresponds to the letter shown in Figure 1B. S/A describes what is the geographical origin of the line (i.e., whether the lines involved in the cross are sympatric or allopatric).
(0.03 MB RTF)

Table S3 **Allopatric and sympatric crosses involving** ***D. santomea*** **females.** Cross corresponds to the letter shown in Figure 1A. S/A describes what is the geographical origin of the line (i.e., whether the lines involved in the cross are sympatric or allopatric).
(0.03 MB RTF)

Table S4 **Degree of sexual isolation for allopatric (A) and sympatric (S) crosses.** S/A describes what is the geographical origin of the line (i.e., whether the lines involved in the cross are sympatric or allopatric). *N*, the number of pairings observed for each mating type equaled 80 in all the cases. In the four mating columns, Y refers to *D. yakuba*, S to *D. santomea*, and the species of the female in each pairing is given first. Ipsi is the proposed statistic by Rolan-Alvarez to measure sexual isolation. SI(yak) and SI(san) are the degree of sexual isolation for *D. yakuba* females only and *D. santomea* females only, respectively. Allopatric (Al) and sympatric females (Sy) from both species showed no significant differences in sexual isolation in any of the three measurements (Ipsi: *F*_1,17_ = 0.3899, *p* = 0.5406; SI_yak_: *F*_1,17_ = 1.554, *p* = 0.2292; SI_san_: *F*_1,17_ = 0.1812, *p* = 0.6757).
(0.08 MB RTF)

Table S5 **Mean (SE) copulation latency in no-choice mating experiments involving** ***D. yakuba*** **females from sympatric and allopatric populations.** *N* is equal to 12 for all crosses. The data were analyzed with a nested ANOVA in which copulation latency was the response. The fixed effects were female line, nested within origin of the *D. yakuba* line (allopatric or sympatric) and male line nested within male origin. Although the female and male line effects caused heterogeneity (Female line: *F*_16,277_ = 10.176, *p* = 2.2×10^−16^; Male line: *F*_7,136_ = 5.011, *p* = 1.22×10^−5^), there was no correlation between copulation latency and whether the populations were sympatric or allopatric (Female origin: *F*_1,99_ = 3.633, *p* = 0.057; Male origin: *F*_1,17_ = 0.6155, *p* = 0.432).
(0.10 MB RTF)

Table S6 **Mean (SE) copulation duration in no-choice mating experiments involving** ***D. yakuba*** **females from sympatric and allopatric populations.** *N* is equal to 12 for all crosses. The data were analyzed with a nested ANOVA in which copulation duration was the response. The fixed effects were female line, nested within origin of the *D. yakuba* line (allopatric or sympatric) and male line nested within male origin. Although the female and male line effects caused heterogeneity (Female line: *F*_16,460_ = 7.407, *p* = 2.2×10^−6^; Male line: *F*_1,230_ = 4.352, *p* = 8.503×10^−5^), there was no correlation between copulation duration and whether the populations were sympatric or allopatric (Female origin: *F*_1,104_ = 1.683, *p* = 0.195; Male origin: *F*_1,230_ = 3.706, *p* = 0.054).
(0.11 MB RTF)

Table S7 **F1 (** ***D. yakuba*** **×** ***D. santomea*** **) larvae survival as a proxy for postzygotic isolation.** Hybrid larvae from matings between females from allopatric or sympatric lines of *D. yakuba* and *D. santomea* were collected as first-instar larvae, and the number of recovered adults was scored. *N* equals 100 for all crosses. The data were analyzed with a nested ANOVA in which the asin (proportion of surviving larvae) was the response. The fixed effects were female line, nested within origin of the *D. yakuba* line (allopatric or sympatric) and male line nested within male origin. Female and male line effects caused heterogeneity (Female line: *F*_4,3589_ = 65.39, *p* = 8.29×10^−16^; Male line: *F*_4,3589_ = 53.011, *p* = 2.12×10^−15^), but there was no correlation between copulation latency and whether the populations were sympatric or allopatric (Female origin: *F*_1,3589_ = 1.84, *p* = 0.1192; Male origin: *F*_1,3589_ = 0.6155, *p* = 0.582).
(0.03 MB RTF)

## Abbreviations

CSP: conspecific sperm precedence
LMM: linear mixed model
