# Supplementary figures and images for: Reinforcement of Gametic Isolation in Drosophila
Source: PLoS Biol. 2010 Mar 23;8(3):e1000341. doi: 10.1371/journal.pbio.1000341 (PMC2843595; doi:10.1371/journal.pbio.1000341)

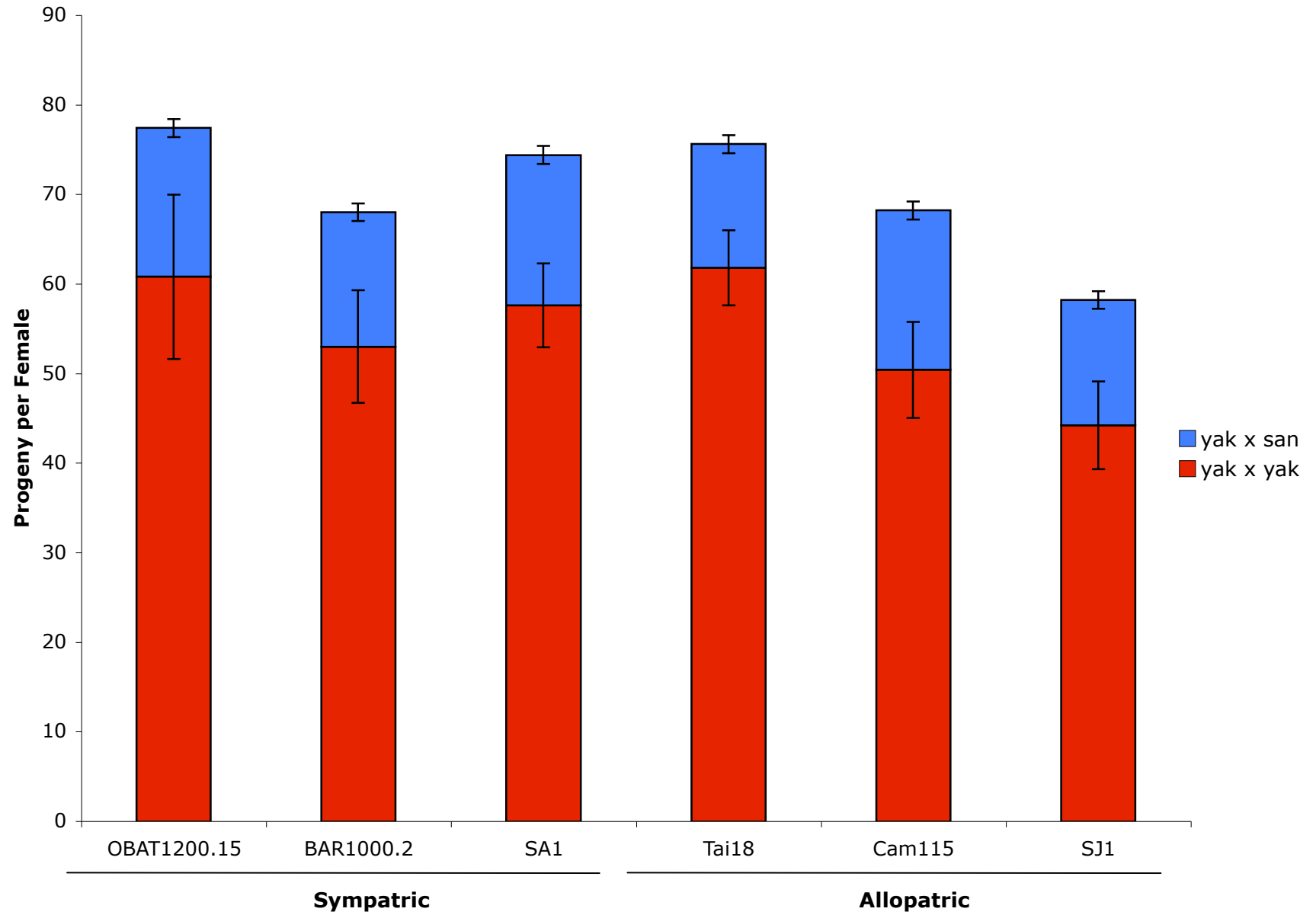

Supplement: Figure S1 — Offspring production from double matings by D. yakuba females I. Mean (SE) number of offspring per D. yakuba female (from either sympatric or allopatric populations) sired by first (D. yakuba, red) and second (D. santomea STO.4, blue) male. The number of offspring produced during the first 4 d was subtracted from the total amount of produced progeny. The data were analyzed with a nested ANOVA in which the asin (progeny produced after the second mating/total progeny) was the response and line was nested within origin of the D. yakuba line (allopatric or sympatric). The results (Female origin: F 1,54 = 0.069, p = 0.794; Female line: F 4,54 = 1.188, p = 0.068) show no difference in the strength of CSP between sympatric and allopatric lines when D. yakuba is the first male. (0.02 MB PDF) [file pbio.1000341.s001.pdf]

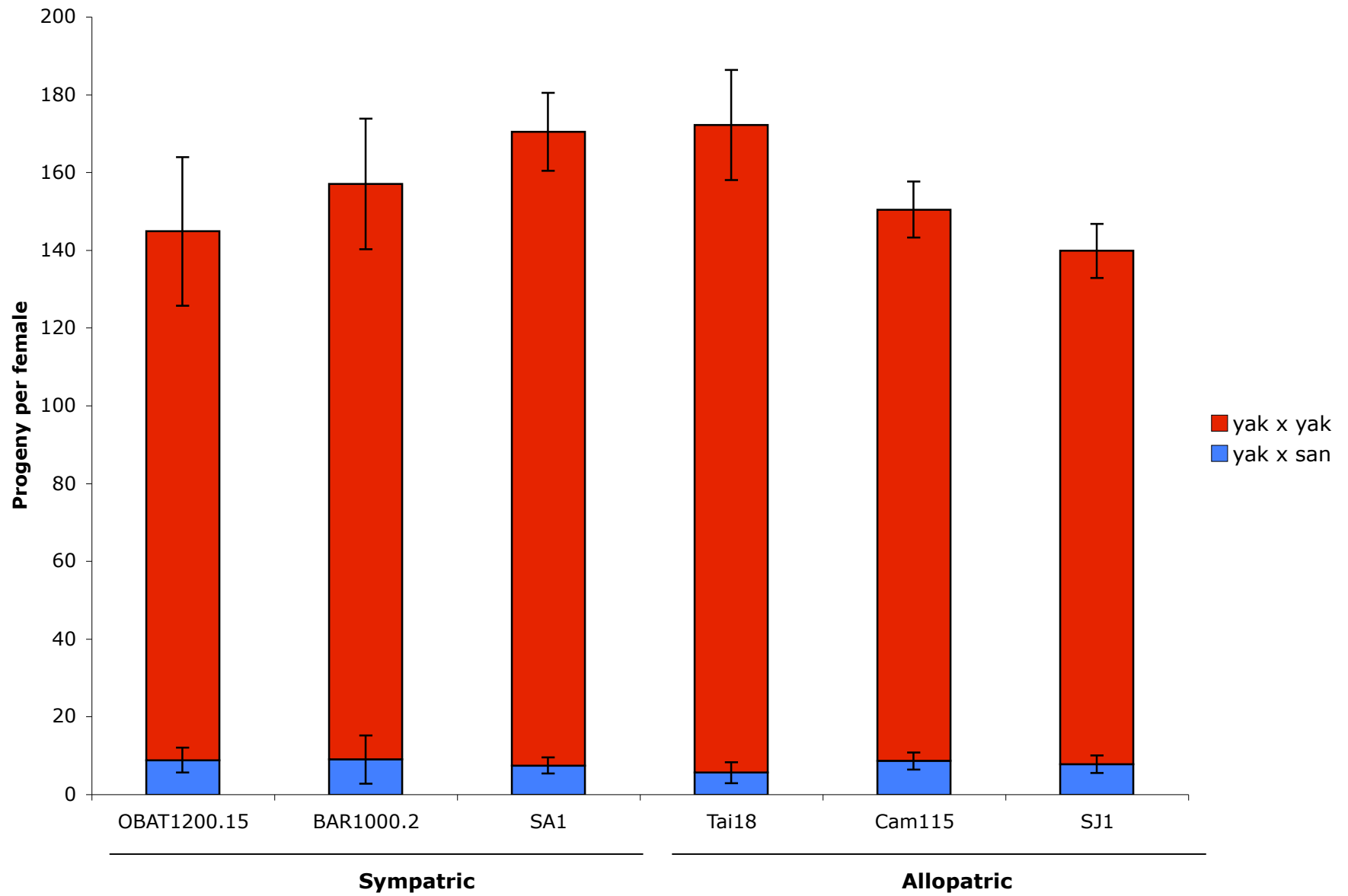

Supplement: Figure S2 — Offspring production from double matings by D. yakuba females II. Mean (SE) number of offspring per D. yakuba female (from either sympatric or allopatric populations) sired by first (D. santomea STO.4, red) and second (D. yakuba, blue) male. The number of offspring produced during the first 4 d was subtracted from the total amount of produced progeny. The data were analyzed with a nested ANOVA in which the asin (progeny produced after the second mating/total progeny) was the response and line was nested within origin of the D. yakuba line (allopatric or sympatric). The results (Female origin: F 1,54 = 0.643; Female line: F 4,54 = 1.188, p = 0.327) show no difference in the strength of CSP between sympatric and allopatric lines when D. santomea is the first male. (0.02 MB PDF) [file pbio.1000341.s002.pdf]

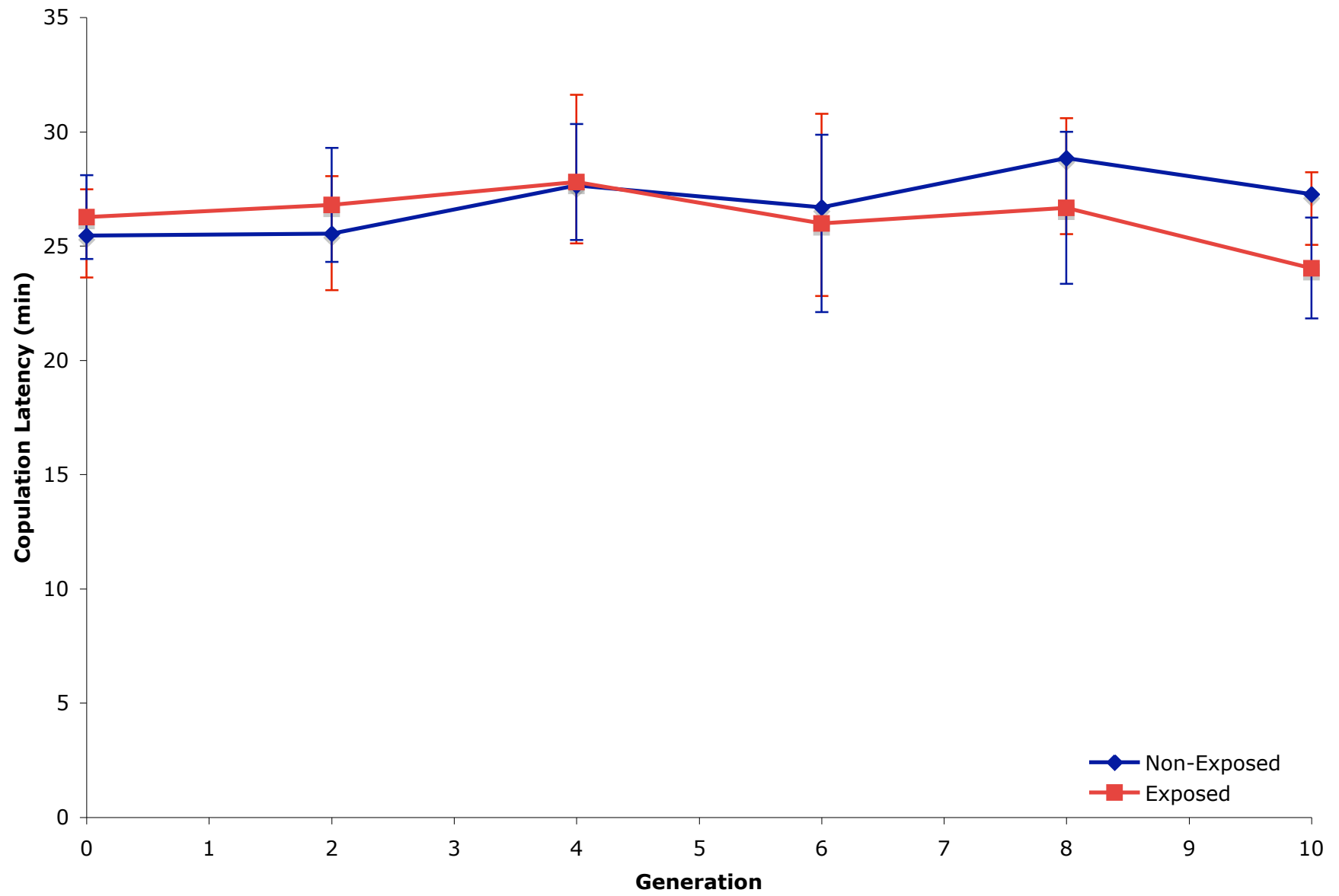

Supplement: Figure S3 — Effects of experimental sympatry on copulation latency in D. yakuba . Means and standard errors are based on the average of the seven lines (four replicates per line). (0.02 MB PDF) [file pbio.1000341.s003.pdf]

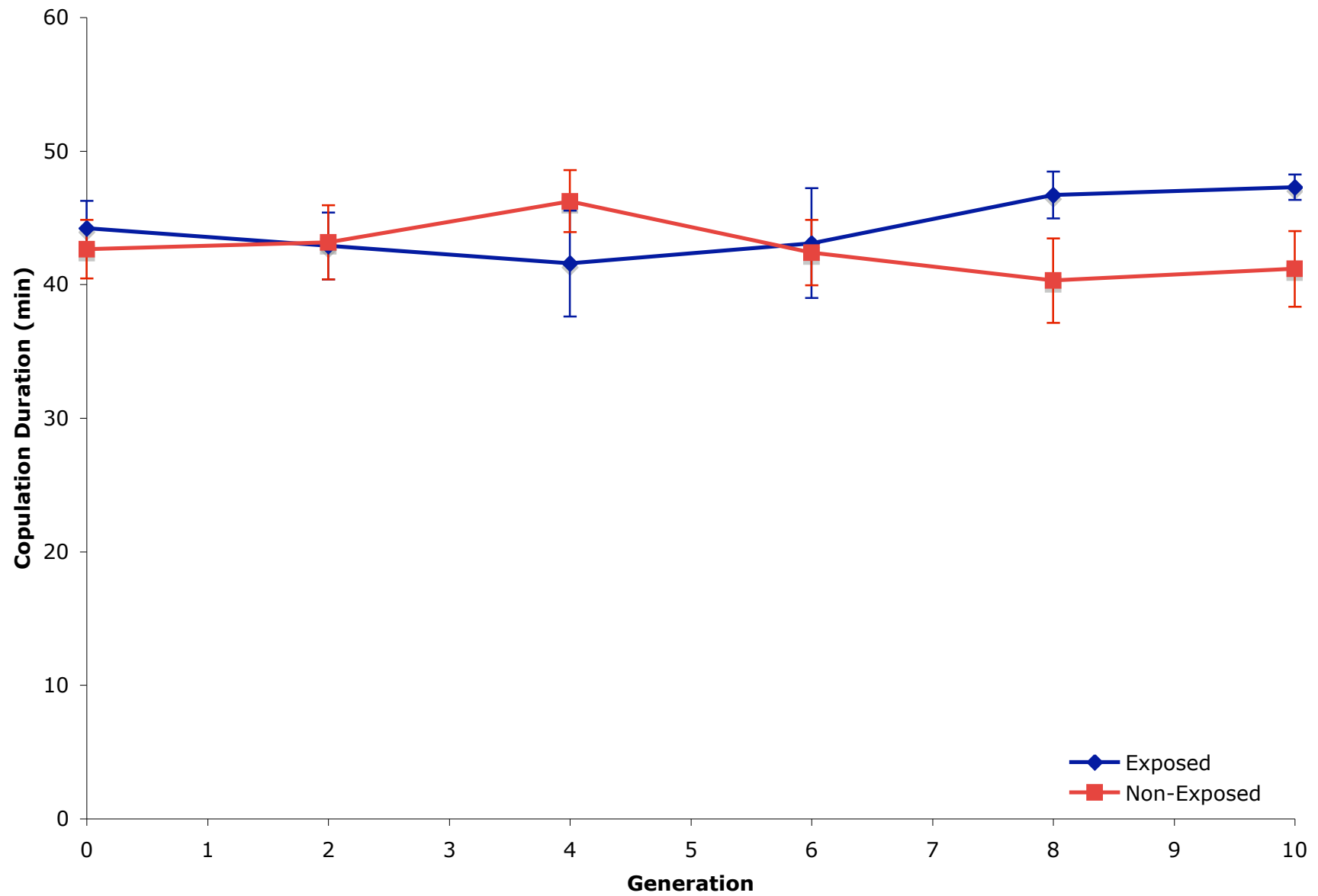

Supplement: Figure S4 — Effects of experimental sympatry on copulation duration in D. yakuba . Means and standard errors are based on the average of the seven lines (four replicates per line). (0.02 MB PDF) [file pbio.1000341.s004.pdf]

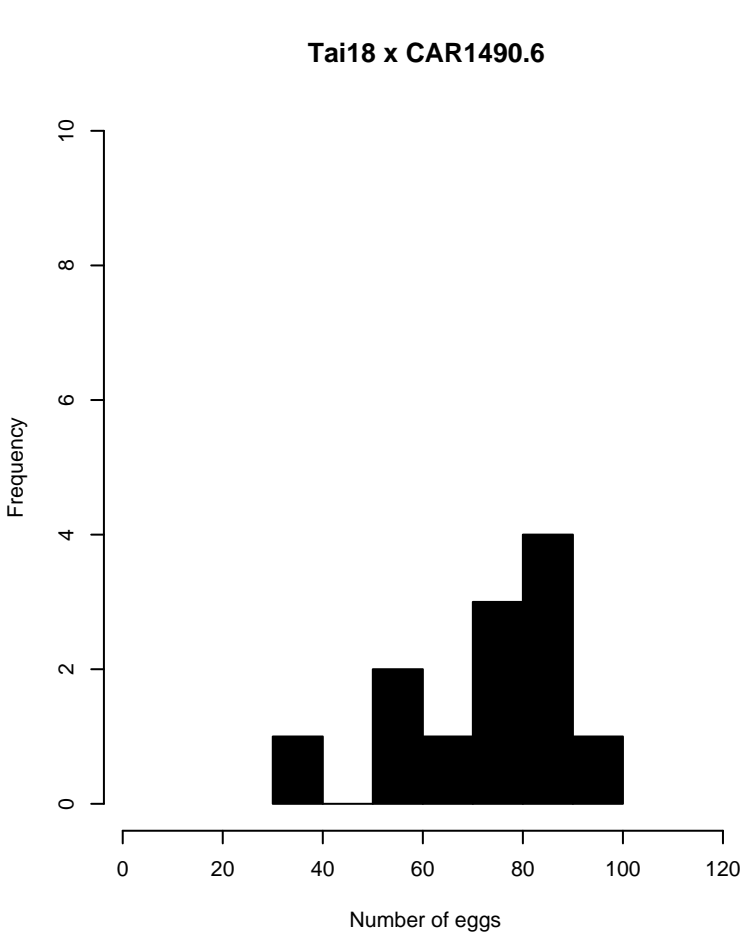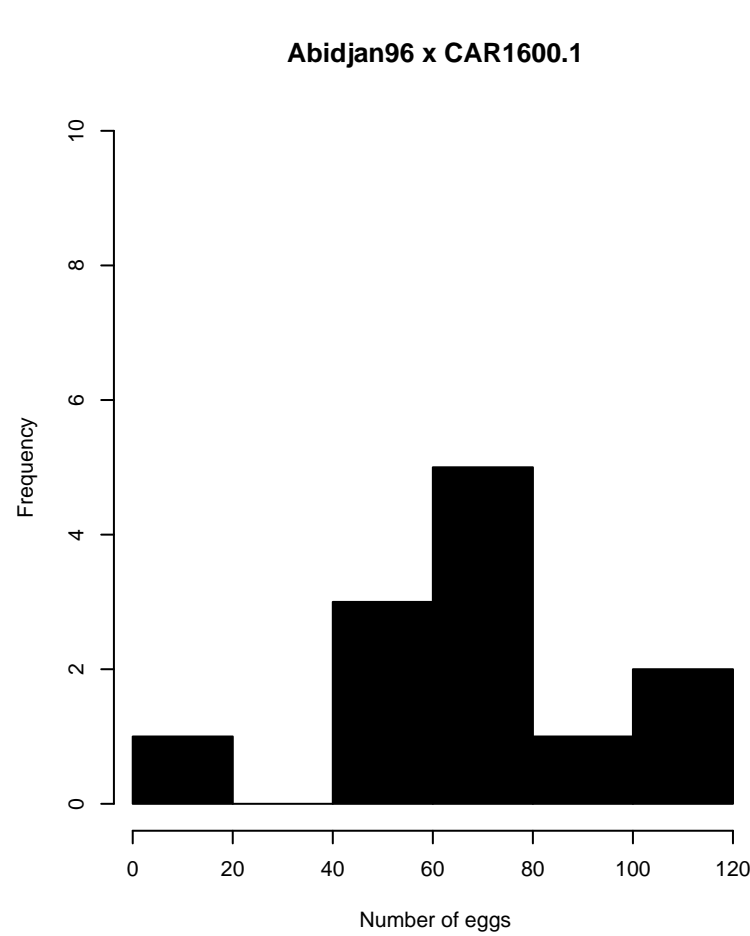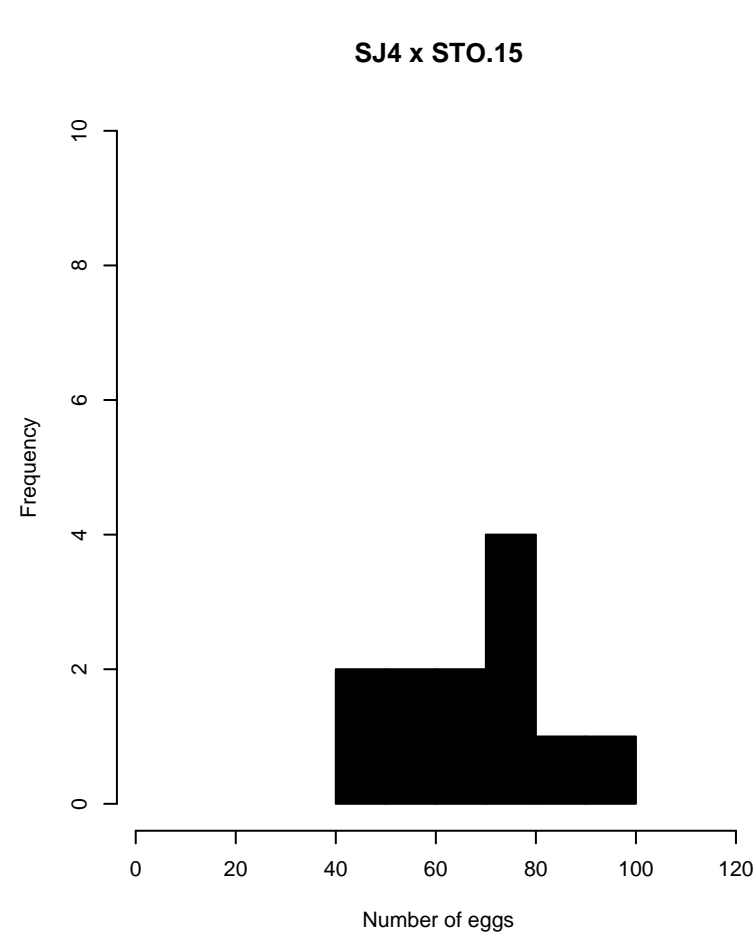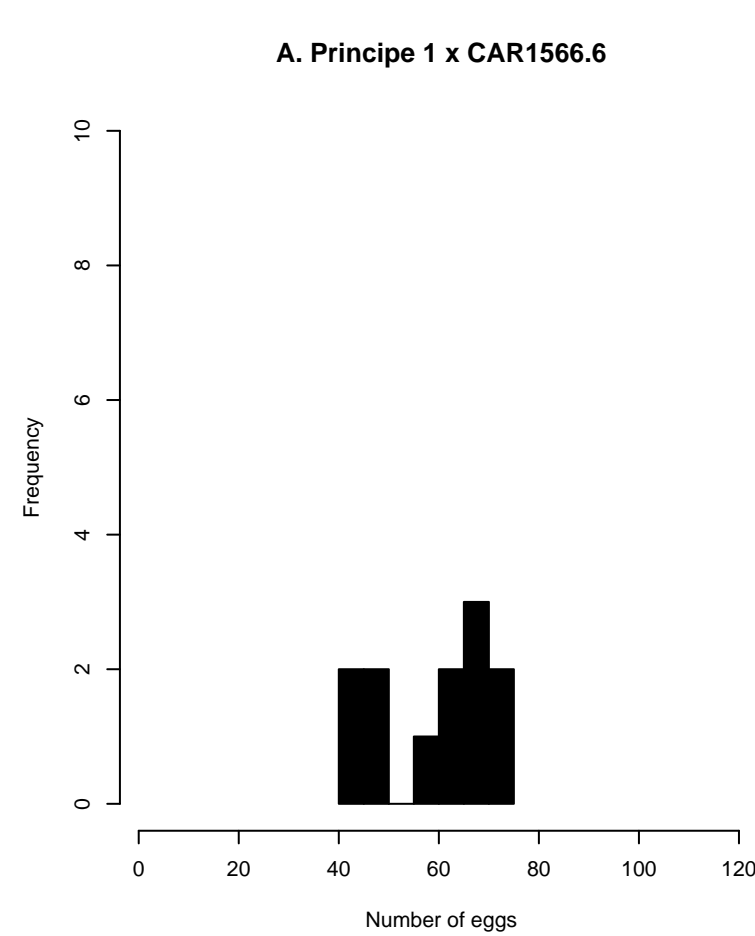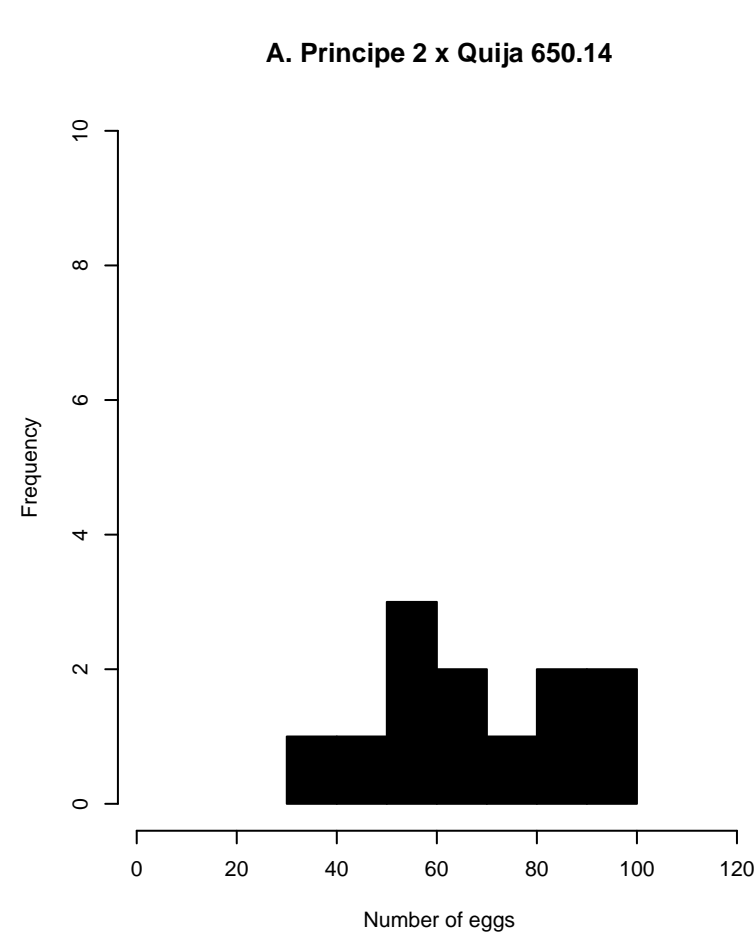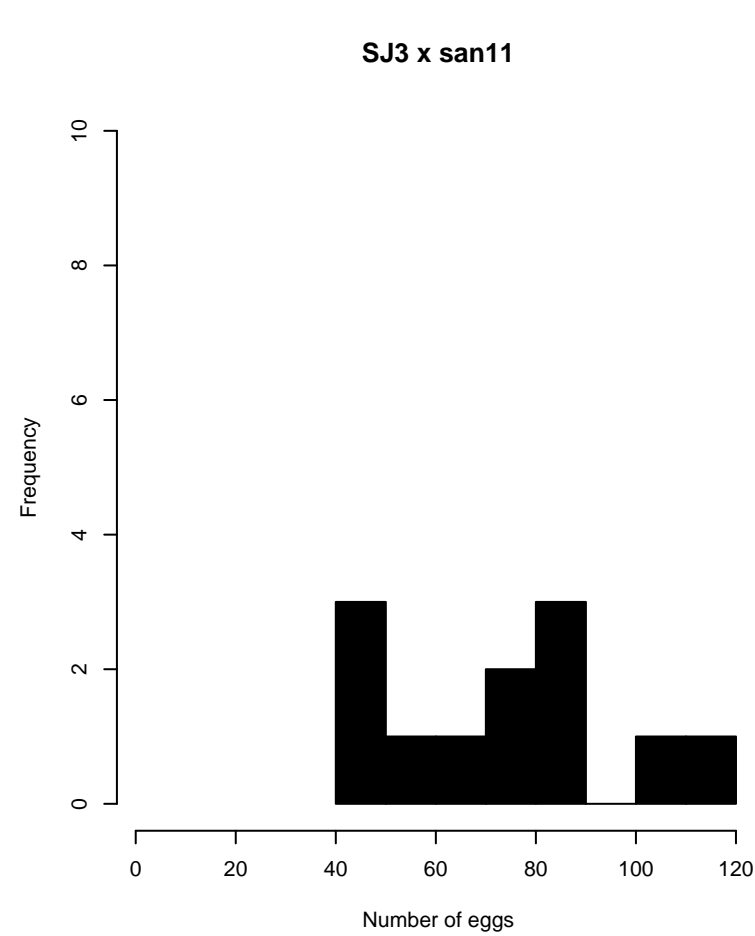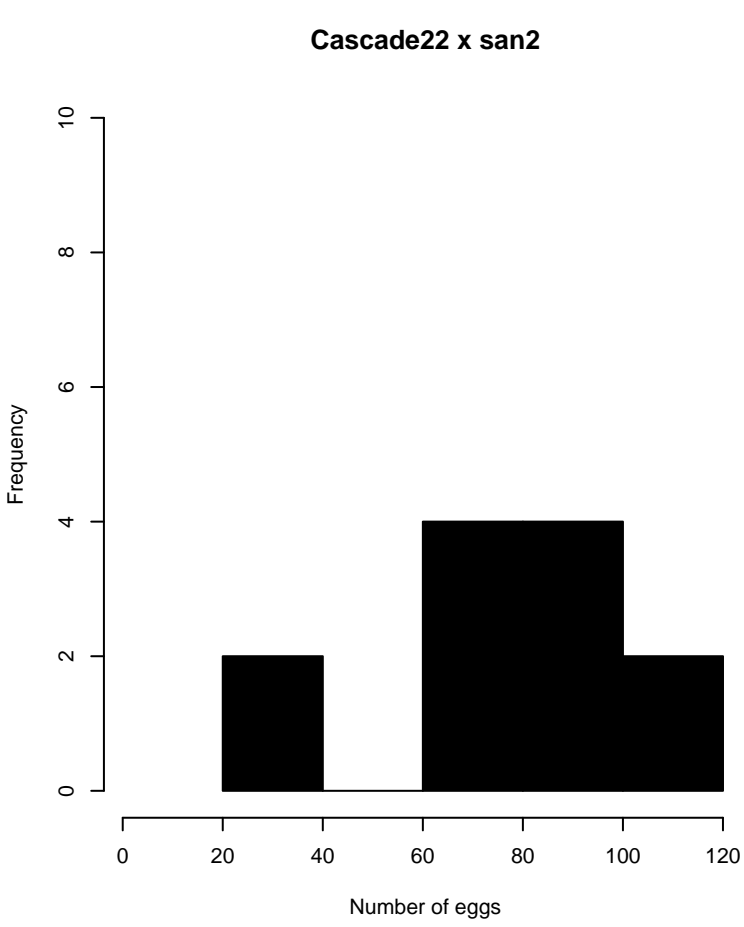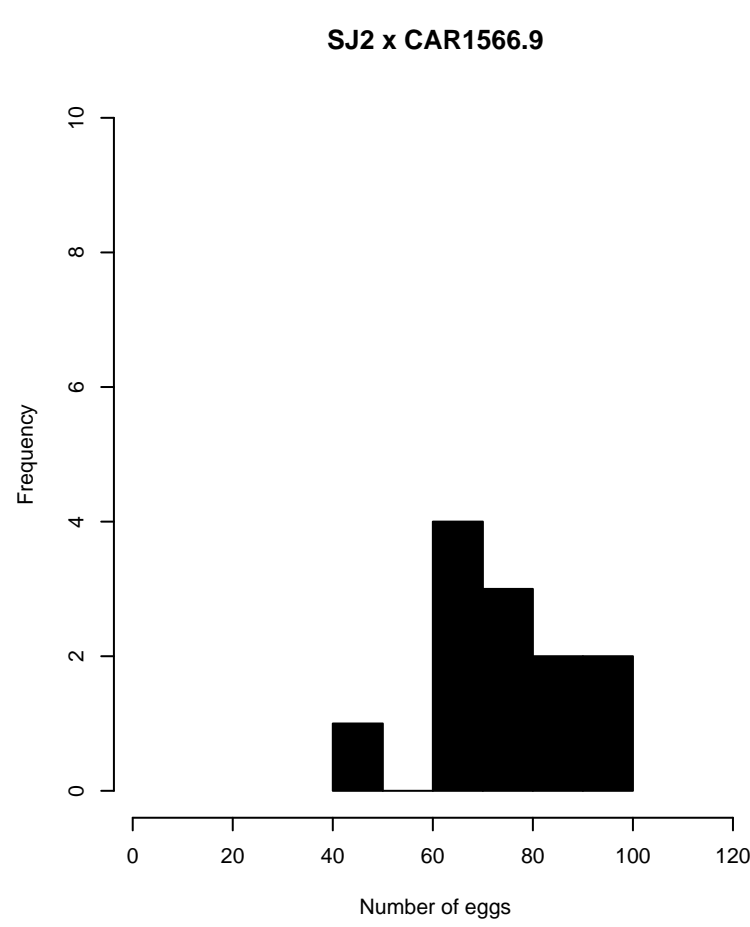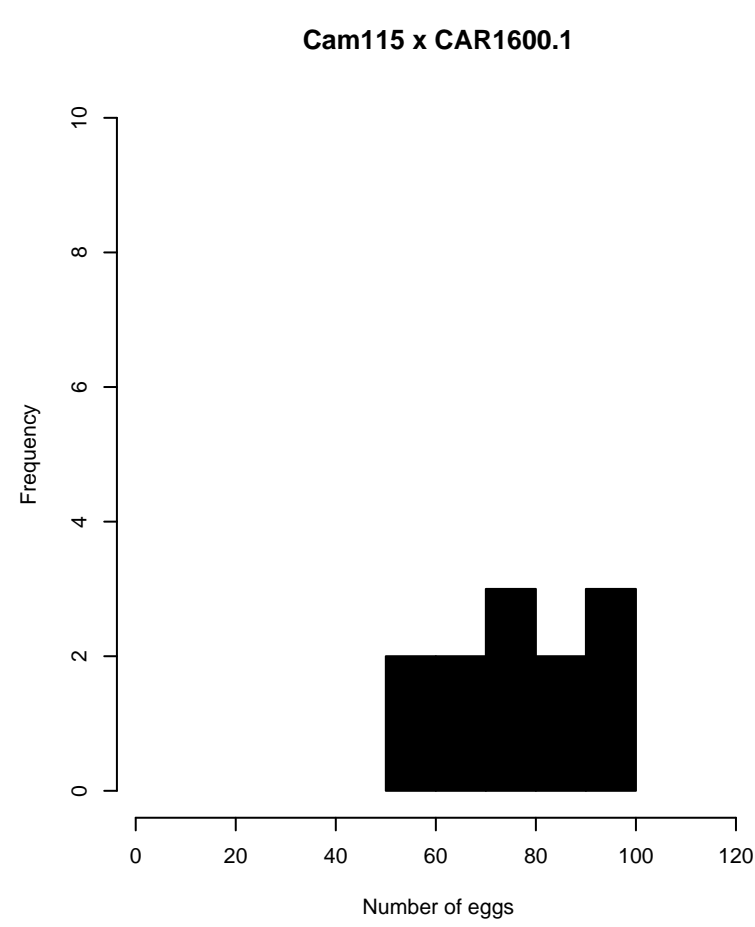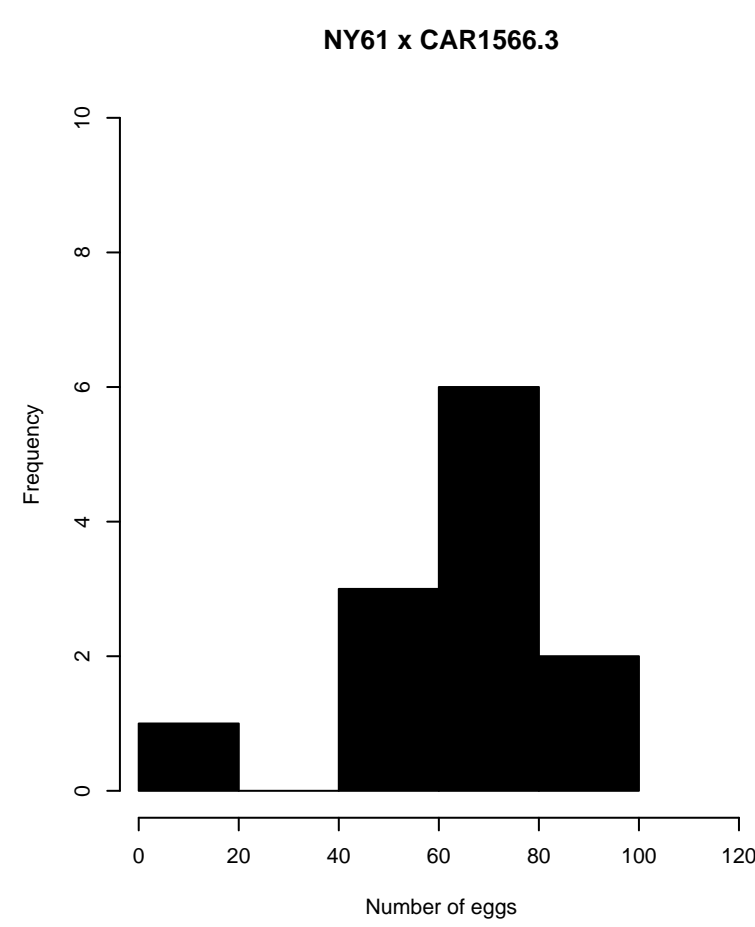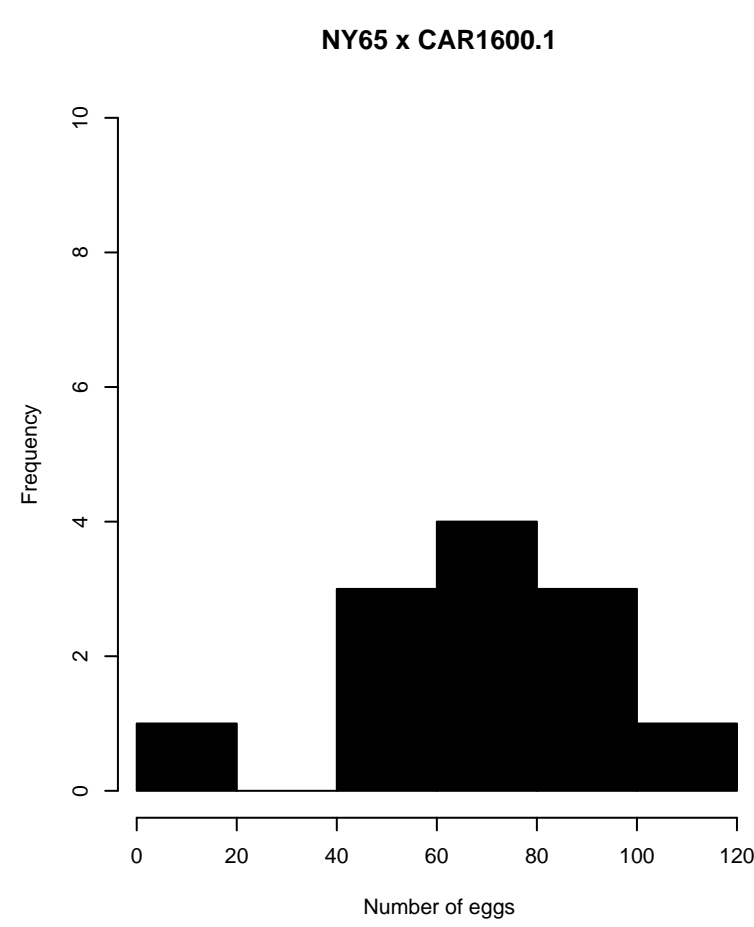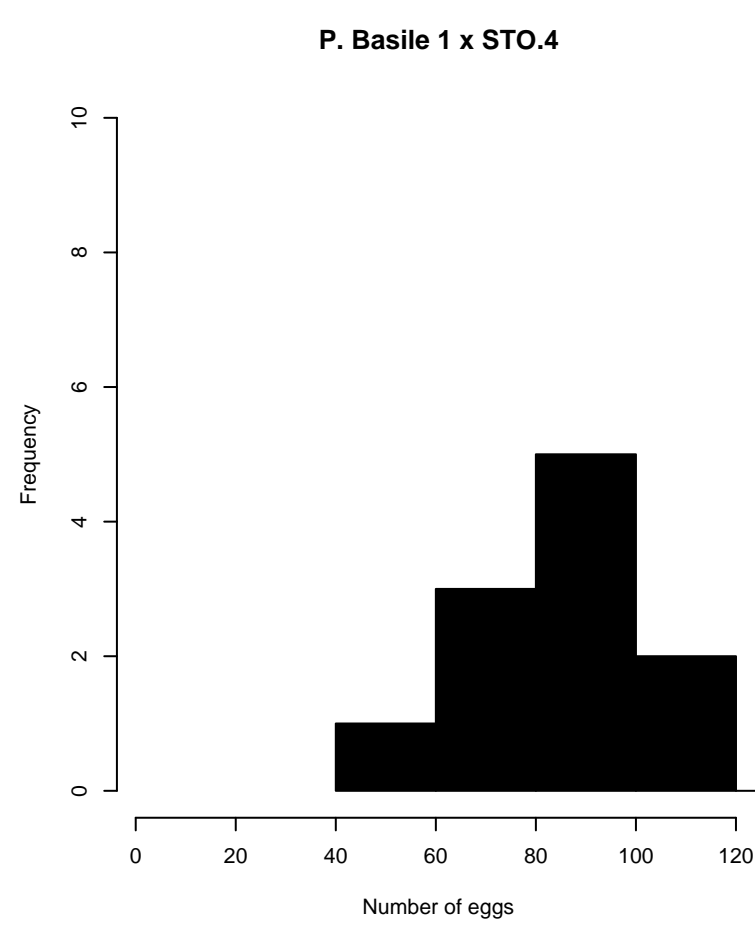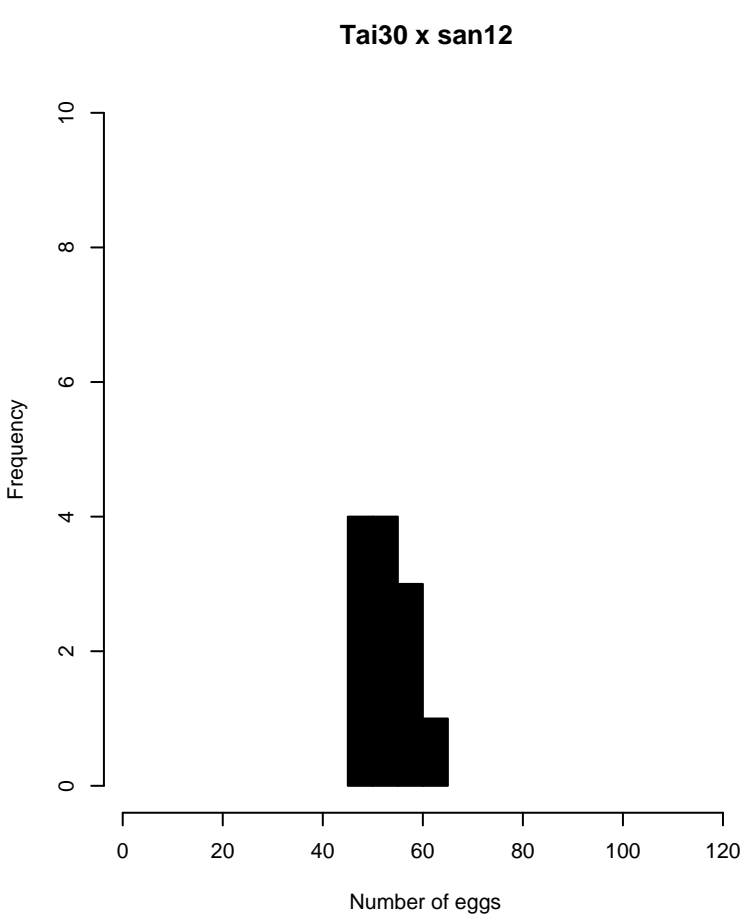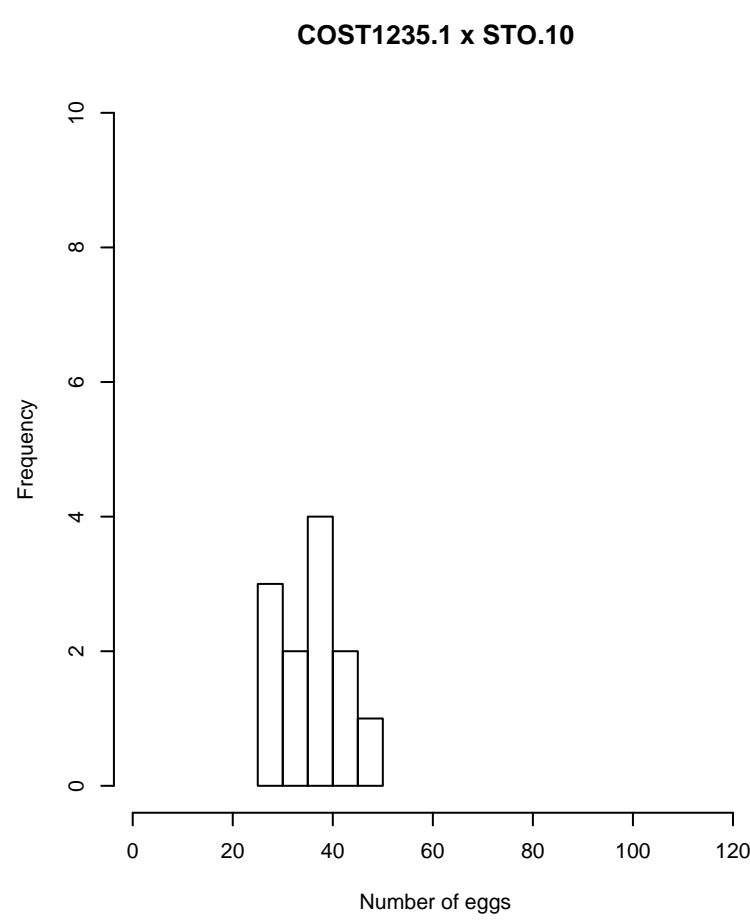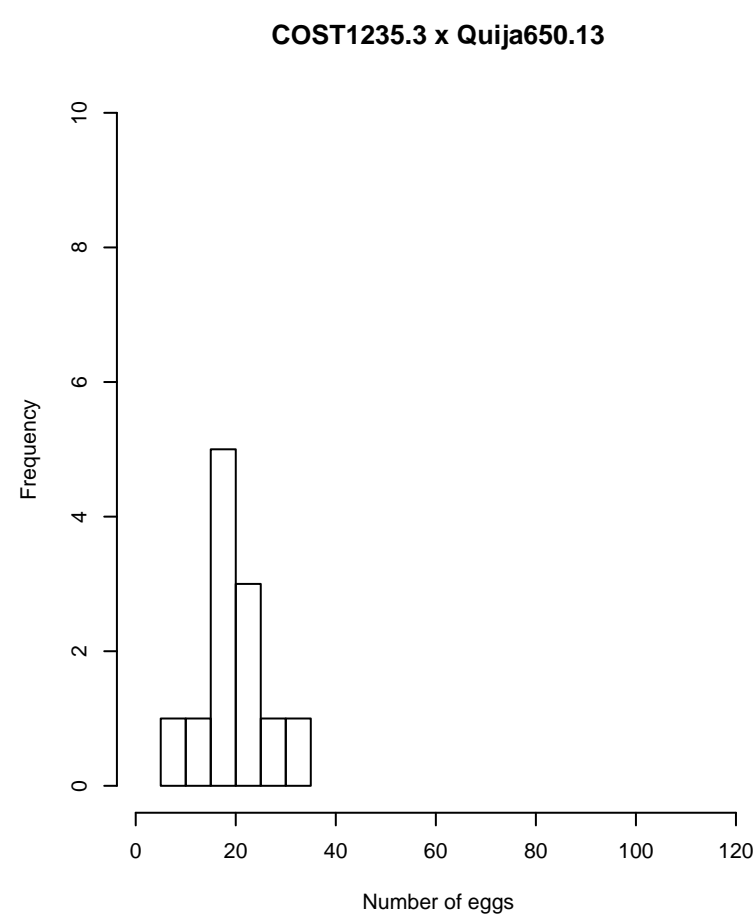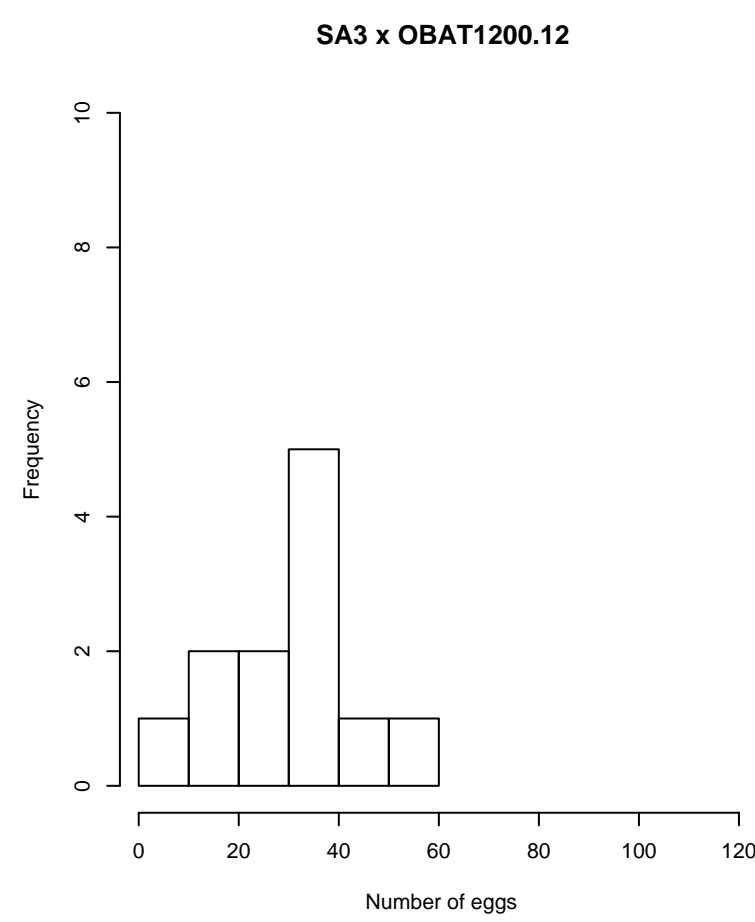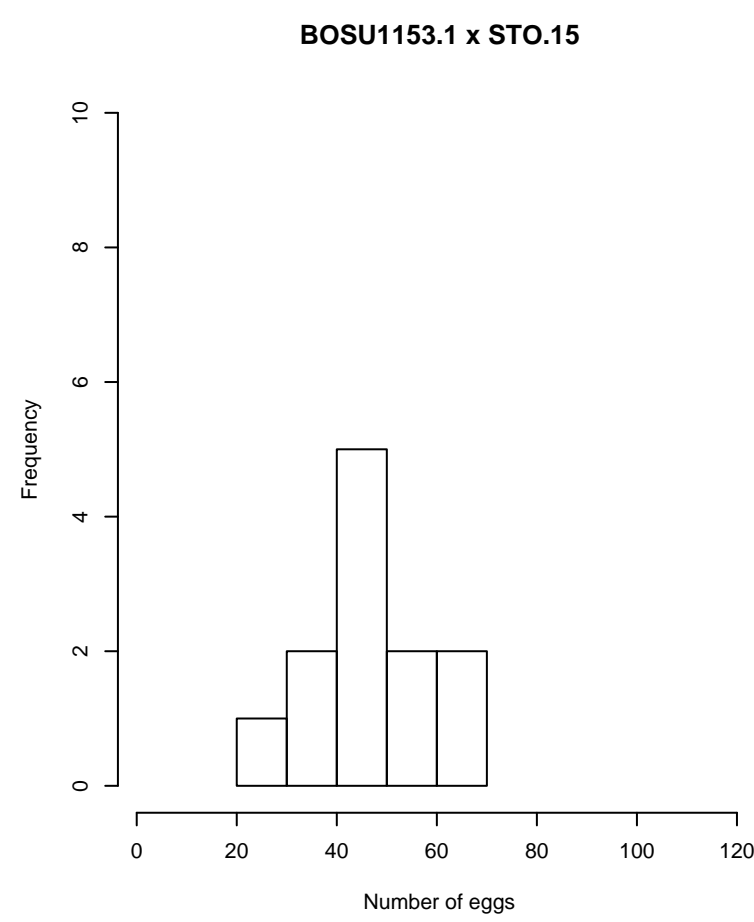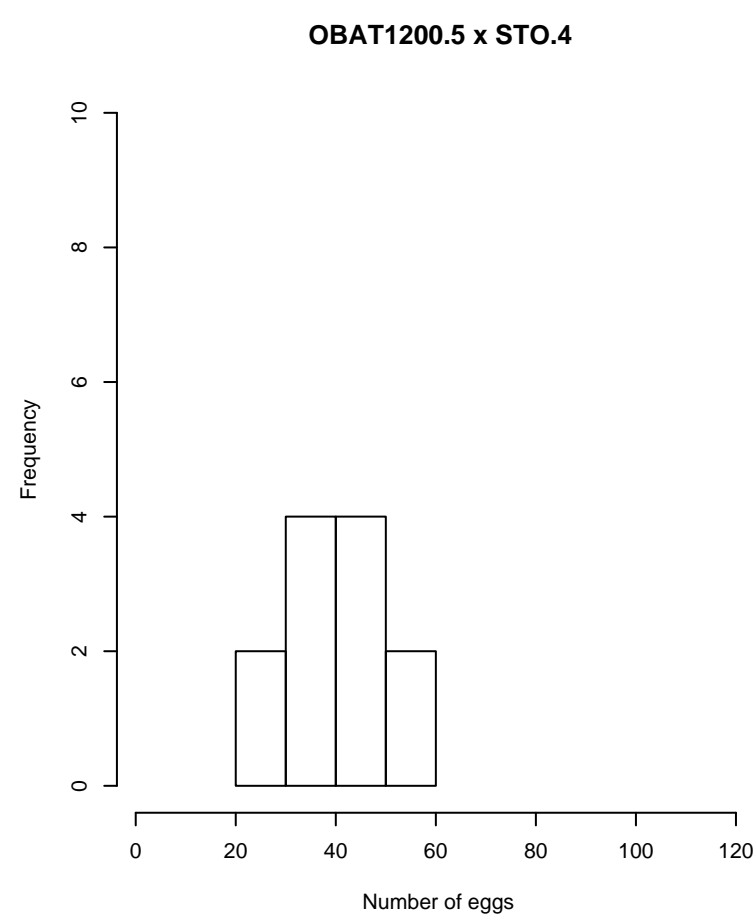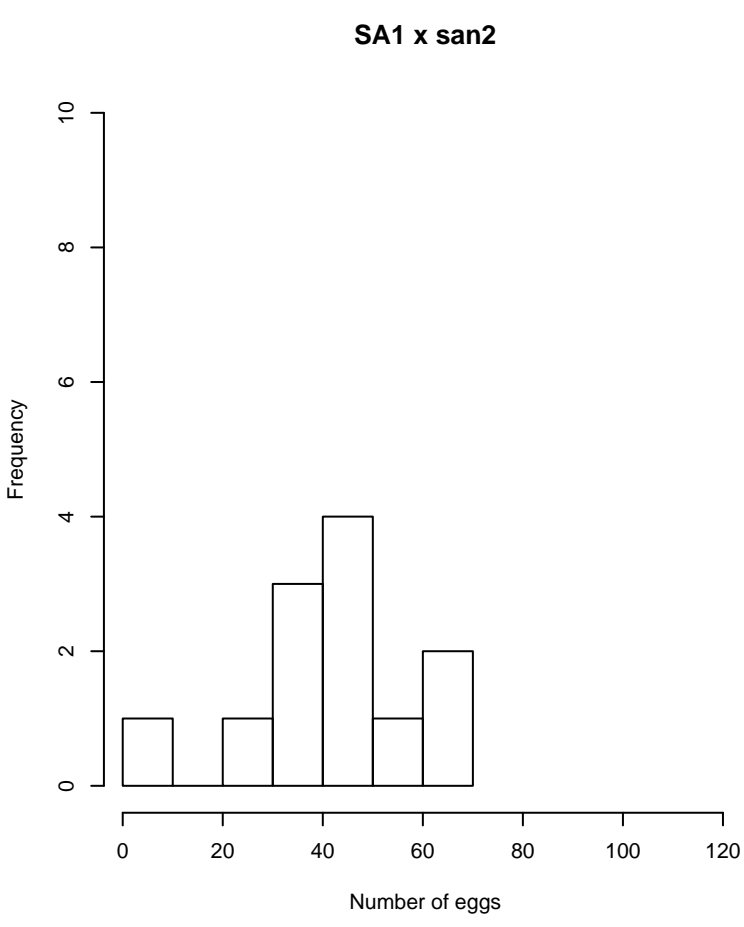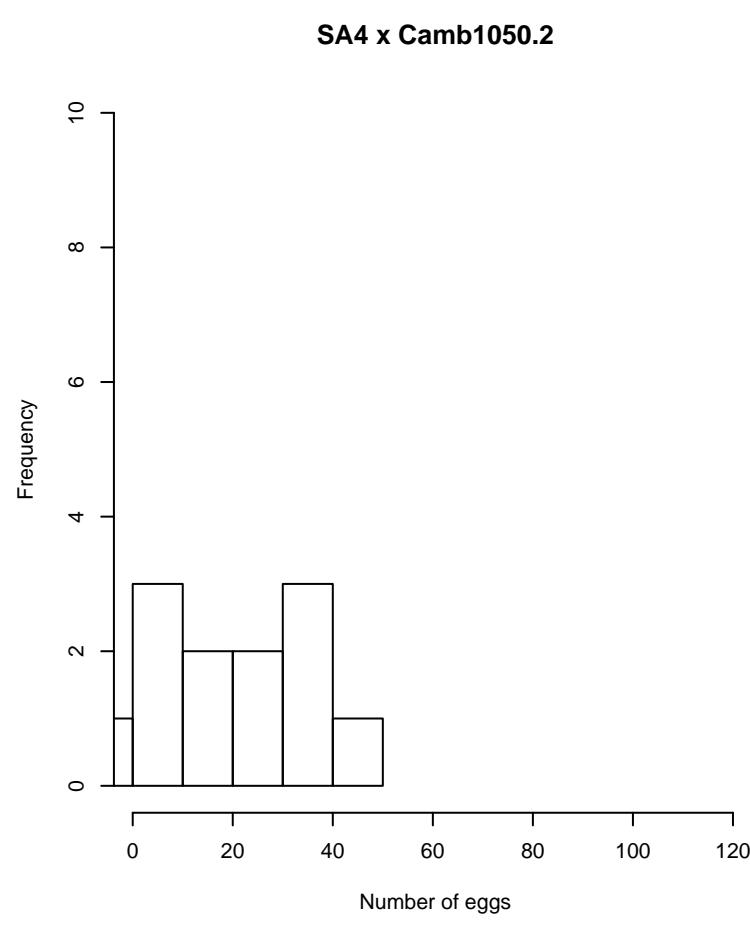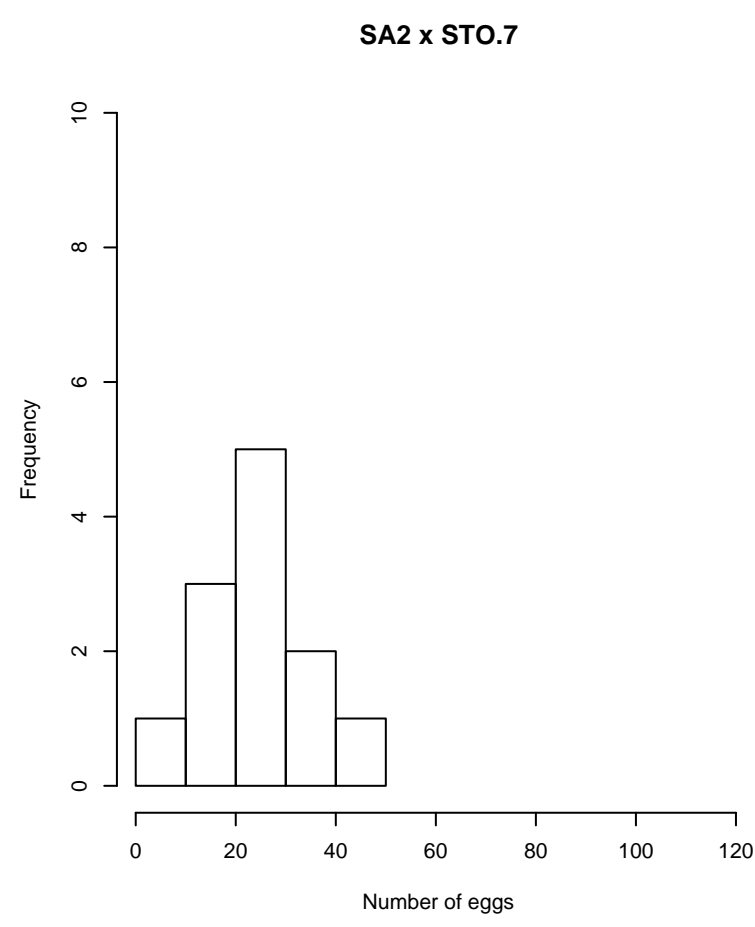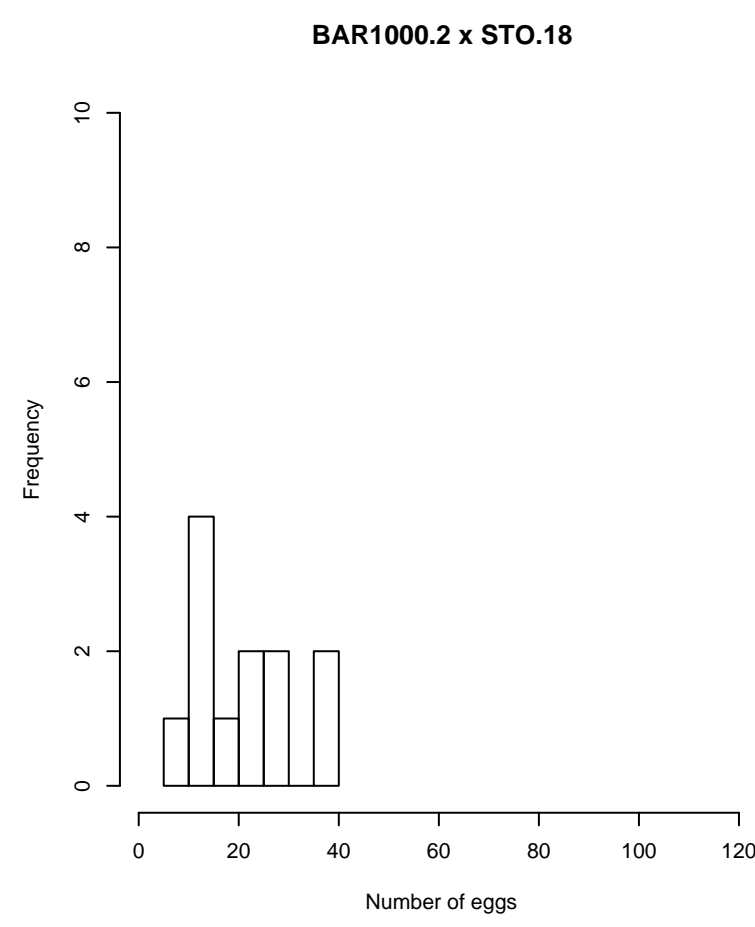

Supplement: Figure S5 — Frequency distributions of gametic isolation levels per D. yakuba line. The title of each graph shows the lines involved in the cross (♀ D. yakuba × ♂ D. santomea). Black distributions: allopatric lines; white distributions: sympatric lines. The data shown in this figure are the same data shown in Figure 1. (0.04 MB PDF) [file pbio.1000341.s005.pdf]
