# Supplementary material for: Reinforcement of Gametic Isolation in Drosophila
Source: PLoS Biol. 2010 Mar 23;8(3):e1000341. doi: 10.1371/journal.pbio.1000341 (PMC2843595; doi:10.1371/journal.pbio.1000341)
Supplement: Table S1 — Isofemale lines of D. santomea and D. yakuba analyzed in this study. (0.10 MB RTF) [file pbio.1000341.s006.rtf]

Supplementary Table 1.

Species	Name	Location	Collector	Date	Allopatric /Sympatric	
D. yakuba	Cameroon 115	Kounden Plateau in western Cameroon 	D. Lachaise	1988 April	Allopatric	
D. yakuba	Täi 18	Tai, Guinea	D. Lachaise	2000 May	Allopatric	
D. yakuba	Täi 30	Tai, Guinea	D. Lachaise	2000 May	Allopatric	
D. yakuba	Abidjan-96	Ivory Coast	Provided by T. MacKay	-	Allopatric	
D. yakuba	Anton-1 Principe	Príncipe, São Tomé and Príncipe	D. Lachaise	-	Allopatric	
D. yakuba	Anton-2 Principe	Príncipe, São Tomé and Príncipe	D. Lachaise	-	Allopatric	
D. yakuba	Cascade 20	Cascade, São Tomé and Príncipe	A. Llopart - Jerry Coyne	2005 January	Allopatric	
D. yakuba	Cascade 22	Cascade, São Tomé and Príncipe	A. Llopart - Jerry Coyne	2005 January	Allopatric	
D. yakuba	SJ1	Sao Joao dos Angolares, São Tomé and Príncipe 	D. Lachaise	2000 March	Allopatric	
D. yakuba	NY61	Nairobi, Kenya	P. Andolfatto	2007	Allopatric	
D. yakuba	NY65	Nairobi, Kenya	P. Andolfatto	2007	Allopatric	
D. yakuba	PB1	Pico Basile, Equatorial Guinea	D. Matute	2009	Allopatric	
D. yakuba	SJ2	Sao Joao dos Angolares, São Tomé and Príncipe 	D. Lachaise	2000 March	Allopatric	
D. yakuba	SJ3	Sao Joao dos Angolares, São Tomé and Príncipe 	D. Lachaise	2000 March	Allopatric	
D. yakuba	SJ4	Sao Joao dos Angolares, São Tomé and Príncipe 	D. Lachaise	2000 March	Allopatric	
D. yakuba	SA.1	Obo Natural Reserve (1150-1350 m), São Tomé and Príncipe	D. Lachaise	January 1998	Sympatric	
D. yakuba	SA.2	Obo Natural Reserve (1150-1350 m), São Tomé and Príncipe	D. Lachaise	January 1998	Sympatric	
D. yakuba	SA.3	Obo Natural Reserve (1150-1350 m), São Tomé and Príncipe	D. Lachaise	January 1998	Sympatric	
D. yakuba	SA.4	Obo Natural Reserve (1150-1350 m), São Tomé and Príncipe	D. Lachaise	January 1998	Sympatric	
D. yakuba	OBAT 1200.5	Obo Natural Reserve (1200 m), São Tomé and Príncipe	D. Lachaise	2001	Sympatric	
D. yakuba	BAR 1000.2	Obo Natural Reserve (1000 m, below hybrid zone), São Tomé and Príncipe	D. Lachaise	2001	Sympatric	
D. yakuba	COST 1235.2	Obo Natural Reserve (1235m), São Tomé and Príncipe	D. Lachaise	2001	Sympatric	
D. yakuba	BOSSU1153.1	Obo Natural Reserve (1153m), São Tomé and Príncipe	D. Lachaise	2005	Sympatric	
D. yakuba	COST  1235.3	Obo Natural Reserve (1235m), São Tomé and Príncipe	D. Lachaise	2001	Sympatric	
D. santomea	STO.4	Obo Natural Reserve (1150-1350 m), São Tomé and Príncipe	D. Lachaise	May 2000	Sympatric	
D. santomea	STO.7	Obo Natural Reserve (1150-1350 m), São Tomé and Príncipe	D. Lachaise	May 2000	Sympatric	
D. santomea	STO.10	Obo Natural Reserve (1150-1350 m), São Tomé and Príncipe	D. Lachaise	May 2000	Sympatric	
D. santomea	STO.15	Obo Natural Reserve (1150-1350 m), São Tomé and Príncipe	D. Lachaise	May 2000	Sympatric	
D. santomea	STO.18	Obo Natural Reserve (1150-1350 m), São Tomé and Príncipe	D. Lachaise	May 2000	Sympatric	
D. santomea	Quija 650.13	Rio Quija, São Tomé and Príncipe	A. Llopart - Jerry Coyne	-	Sympatric	
D. santomea	Quija 650.14	Rio Quija, São Tomé and Príncipe	A. Llopart - Jerry Coyne	-	Sympatric	
D. santomea	Cambúmbe 1050.2	Rio Quija, São Tomé and Príncipe	A. Llopart - Jerry Coyne	-	Sympatric	
D. santomea	CAR.1600.1	Pico do Carvalho, São Tomé and Príncipe 	A. Llopart - Jerry Coyne	January 2001	Allopatric	
D. santomea	CAR.1566.3	Pico do Carvalho, São Tomé and Príncipe 	A. Llopart - Jerry Coyne	January 2001	Allopatric	
D. santomea	CAR.1566.9	Pico do Carvalho, São Tomé and Príncipe 	A. Llopart - Jerry Coyne	January 2001	Allopatric	
D. santomea	CAGO1495.5	Pico do Carvalho, São Tomé and Príncipe 	A. Llopart - Jerry Coyne	January 2001	Allopatric	
D. santomea	CAR.1455.6	Pico do Carvalho, São Tomé and Príncipe 	A. Llopart - Jerry Coyne	January 2001	Allopatric	
D. santomea	CAR.1600.3	Pico do Carvalho, São Tomé and Príncipe 	A. Llopart - Jerry Coyne	January 2001	Allopatric	
D. santomea	1	Bom Successo, São Tomé and Príncipe  	A. Llopart - Jerry Coyne	January 2005	Sympatric	
D. santomea	2	Bom Successo, São Tomé and Príncipe  	A. Llopart - Jerry Coyne	January 2005	Sympatric	
D. santomea	12	Bom Successo, São Tomé and Príncipe  	A. Llopart - Jerry Coyne	January 2005	Sympatric	
