# Supplementary material for: Reinforcement of Gametic Isolation in Drosophila
Source: PLoS Biol. 2010 Mar 23;8(3):e1000341. doi: 10.1371/journal.pbio.1000341 (PMC2843595; doi:10.1371/journal.pbio.1000341)
Supplement: Table S2 — Allopatric and sympatric crosses involving D. yakuba females. Cross corresponds to the letter shown in Figure 1B. S/A describes what is the geographical origin of the line (i.e., whether the lines involved in the cross are sympatric or allopatric). (0.03 MB RTF) [file pbio.1000341.s007.rtf]

Supplementary Table 2. 
 Cross	D. yakuba female	D. santomea male	Allopatric/Sympatric	
A	SJ2	CAR1566.9	A	
B	SJ3	san1	A	
C	Täi18	CAR1490.6	A	
D	Abidjan 96	CAR1600.1	A	
E	Cascade22  	CAGO1495.5	A	
F	SJ4	STO.15	A	
G	Täi30	san12	A	
H	Anton 1 Principe	CAR1566.6	A	
I	Anton 2 Principe	Quija 650.17	A	
J	Cameroon 115	CAR1600.3	A	
K	NY68	STO.4	A	
L	NY61	CAR1566.9	A	
M	PB1	CAR1600.1	A	
N	COST1235.1	STO.10	S	
O	SA3	OBAT1200.13	S	
P	BOSU1153.1	STO.15	S	
Q	OBAT1200.5	STO.4	S	
R	SA1	san2	S	
S	SA4	Cambúmbe 1050.2	S	
T	SA2	STO.7	S	
U	COST1235.3	Quija 650.13	S	
V	BAR1000.2	STO.18	S	
