# Supplementary material for: Reinforcement of Gametic Isolation in Drosophila
Source: PLoS Biol. 2010 Mar 23;8(3):e1000341. doi: 10.1371/journal.pbio.1000341 (PMC2843595; doi:10.1371/journal.pbio.1000341)
Supplement: Table S3 — Allopatric and sympatric crosses involving D. santomea females. Cross corresponds to the letter shown in Figure 1A. S/A describes what is the geographical origin of the line (i.e., whether the lines involved in the cross are sympatric or allopatric). (0.03 MB RTF) [file pbio.1000341.s008.rtf]

Supplementary Table 3. 
 Cross	D. santomea female	D. yakuba male	Allopatric/Sympatric	
A	CAR1600.1	SJ3	A	
B	CAR1566.6	Anton 1 Principe	A	
C	CAR1490.6	Täi30	A	
D	CAR1600.3	SJ1	A	
E	CAGO1495.5	SJ4	A	
F	CAR1566.9	Cascade22  	A	
G	STO.7	SA2	S	
H	Quija 650.13	SA1	S	
I	San13	COST1235.1	S	
J	STO.18	SA4	S	
K	STO.15	COST1235.3	S	
L	Quija 650.14	BAR1000.2	S	
M	Quija 650.13	OBAT1200.5	S	
N	STO.4	SA3	S	
O	OBAT1200.12	BOSU1153.1	S	
