# Supplementary material for: Reinforcement of Gametic Isolation in Drosophila
Source: PLoS Biol. 2010 Mar 23;8(3):e1000341. doi: 10.1371/journal.pbio.1000341 (PMC2843595; doi:10.1371/journal.pbio.1000341)
Supplement: Table S4 — Degree of sexual isolation for allopatric (A) and sympatric (S) crosses. S/A describes what is the geographical origin of the line (i.e., whether the lines involved in the cross are sympatric or allopatric). N, the number of pairings observed for each mating type equaled 80 in all the cases. In the four mating columns, Y refers to D. yakuba, S to D. santomea, and the species of the female in each pairing is given first. Ipsi is the proposed statistic by Rolan-Alvarez to measure sexual isolation. SI(yak) and SI(san) are the degree of sexual isolation for D. yakuba females only and D. santomea females only, respectively. Allopatric (Al) and sympatric females (Sy) from both species showed no significant differences in sexual isolation in any of the three measurements (Ipsi: F 1,17 = 0.3899, p = 0.5406; SIyak: F 1,17 = 1.554, p = 0.2292; SIsan: F 1,17 = 0.1812, p = 0.6757). (0.08 MB RTF) [file pbio.1000341.s009.rtf]

Supplementary Table 4. 

 	 	 	 	Mating	SI	SI (yak)	SI (san)	
Cross (D. yakuba x D. santomea)
 	Al/Sy	N	Y X Y	Y X S	S X Y	S X S	Ipsi	SD	t	 	 	
Tai18	CAR1490.6	Al	80	58	31	12	35	0.4	0.08	5.02	0.4655	0.6571	
Abidjan 96	CAR1600.1	Al	80	43	24	1	32	0.64	0.08	8.25	0.4419	0.9688	
Anton 1 Principe	CAR1566.6	Al	80	45	15	10	43	0.56	0.08	7.23	0.6667	0.7674	
Anton 2 Principe	Quija 650.17	Al	80	52	35	8	56	0.5	0.07	7.12	0.3269	0.8571	
Cam115	CAR1600.3	Al	80	48	13	7	42	0.64	0.07	8.83	0.7292	0.8333	
SJ3	san1	Al	80	59	53	4	49	0.5	0.07	7.38	0.1017	0.9184	
Cascade22  	san2	Al	80	37	20	8	31	0.45	0.09	4.91	0.4595	0.7419	
Tai30	san12	Al	80	63	29	5	41	0.58	0.07	8.32	0.5397	0.8780	
SJ2	CAR1566.6	Al	80	44	24	12	52	0.47	0.08	6.14	0.4545	0.7692	
SA3	OBAT1200.13	Sy	80	54	18	13	26	0.42	0.09	4.93	0.6667	0.5000	
COST1235.1	STO.10	Sy	80	59	12	15	57	0.62	0.07	9.54	0.7966	0.7368	
COST1235.3	Quija 650.13	Sy	80	66	12	14	40	0.6	0.07	8.62	0.8182	0.6500	
SA2	STO.7	Sy	80	55	26	4	39	0.59	0.07	8.19	0.5273	0.8974	
OBAT1200.5	STO.4	Sy	80	60	31	11	61	0.52	0.07	7.75	0.4833	0.8197	
SA4	Cambumbe 1050.2	Sy	80	47	19	0	13	0.7	0.08	9.04	0.5957	1.0000	
SA1	san2	Sy	80	43	24	9	32	0.42	0.09	4.87	0.4419	0.7188	
SA3	OBAT1200.12	Sy	80	56	16	0	21	0.74	0.07	10.74	0.7143	1.0000	
BAR1000.2	STO.18	Sy	80	46	32	12	37	0.35	0.08	4.21	0.3043	0.6757	
BOSU1153.1	STO.15	Sy	80	38	25	3	54	0.62	0.07	8.61	0.3421	0.9444	
