# Supplementary material for: Reinforcement of Gametic Isolation in Drosophila
Source: PLoS Biol. 2010 Mar 23;8(3):e1000341. doi: 10.1371/journal.pbio.1000341 (PMC2843595; doi:10.1371/journal.pbio.1000341)
Supplement: Table S5 — Mean (SE) copulation latency in no-choice mating experiments involving D. yakuba females from sympatric and allopatric populations. N is equal to 12 for all crosses. The data were analyzed with a nested ANOVA in which copulation latency was the response. The fixed effects were female line, nested within origin of the D. yakuba line (allopatric or sympatric) and male line nested within male origin. Although the female and male line effects caused heterogeneity (Female line: F 16,277 = 10.176, p = 2.2×10^−16^; Male line: F 7,136 = 5.011, p = 1.22×10^−5^), there was no correlation between copulation latency and whether the populations were sympatric or allopatric (Female origin: F 1,99 = 3.633, p = 0.057; Male origin: F 1,17 = 0.6155, p = 0.432). (0.10 MB RTF) [file pbio.1000341.s010.rtf]

Supplementary Table 5. 


 	 	Female	
 	 	COST1235.1	COST1235.3	Anton 2 Principe	BAR1000.2	Cascade 6	
 
 
 
 
Male
 
 
 
 	STO.10	33.800 (1.769)	20.067 (3.715)	20.792 (3.304)	36.542 (0.187)	36.717 (0.347)	
	STO.4	28.658 (7.072)	51.917 (2.307)	42.600 (3.103)	37.792 (8.509)	28.542 (4.025)	
	STO.7	36.300 (6.015)	31.933 (0.054)	24.742 (5.039)	38.042 (2.452)	32.483 (2.640)	
	san13	18.042 (1.398)	41.058 (5.304)	29.767 (5.334)	34.375 (4.385)	37.158 (1.936)	
	san1	19.125 (2.500)  	33.733 (3.987)	40.733 (7.893)	40.733 (4.327)	22.625 (4.379)	
	CAR1555.1	25.325 (2.126)	35.067 (1.225)	22.567 (2.198)	50.967 (3.178)	30.202 (1.519)	
	CAR1600.1	24.675 (0.487)	18.933 (1.014)	24.792 (3.814)	41.667 (0.333)	20.042 (0.391)	
	CAR1600.3	38.592 (2.528)	29.683 (5.294)	40.633 (3.470)	35.125 (9.298)	20.608 (2.058)	
	STO.18	29.733 (1.675)	25.967 (3.389)	36.875 (6.338)	38.208 (4.610)	29.342 (7.066)	


(continuation)

 	 	Female	
 	 	Cascade 7	BOSU1153.1	SJ1	SJ2	SJ3	
 
 
 
 
Male
 
 
 
 	STO.10	19.017 (2.443)	44.400 (0.014)	27.608 (0.786)	36.008 (3.087)	28.375 (1.099)	
	STO.4	35.983 (7.900)	22.042 (2.358)	41.467 (6.341)	26.317 (3.742)	29.433 (0.595)	
	STO.7	36.625 (3.821)	37.967 (7.491)	23.692 (2.970)	32.163 (3.710)	31.731 (2.141)	
	san13	34.408 (2.017)	34.575 (2.990)	38.022 (1.154)	32.583 (5.277)	26.721 (3.449)	
	san1	40.242 (4.521)	49.108 (2.943)	31.997 (3.225)	39.586 (1.139)	39.058 (4.576)	
	CAR1555.1	38.600 (3.014)	17.442 (0.888)	29.238 (2.848)	26.842 (5.699)	32.898 (2.065)	
	CAR1600.1	32.608 (7.046)	41.758 (2.358)	31.408 (5.671)	22.525 (1.715)	41.100 (2.126)	
	CAR1600.3	44.558 (4.970)	38.433 (7.655)	22.083 (2.591)	30.396 (1.198)	39.158 (8.094)	
	STO.18	39.002 (1.926)	21.383 (2.497)	36.417 (3.991)	32.217 (3.885)	32.431 (3.167)	


(continuation)

		Female	
		SJ4	SA1	SA2	SA3	SA4	
 
 
 
Male
 
 
 
 	STO.10	42.750 (5.396)	41.667 (0.333)	28.883 (0.265)	41.053 (2.343)	25.091 (2.465)	
	STO.4	16.892 (1.983)	35.467 (6.729)	31.508 (1.228)	31.983 (0.095)	32.725 (2.950)	
	STO.7	32.860 (2.669)	35.125 (5.298)	46.158 (3.657)	39.914 (3.089)	28.243 (7.843)	
	san13	34.392 (3.551)	43.258 (5.229)	40.592 (4.027)	34.483 (3.422)	31.121 (3.767)	
	san1	34.294 (1.443)	32.075 (5.828)	30.396 (6.155)	26.951 (2.916)	35.625 (2.119)	
	CAR1555.1	19.683 (2.558)	33.692 (9.318)	38.355 (2.956)	31.938 (2.627)	33.351 (2.364)	
	CAR1600.1	25.908 (3.860)	31.883 (8.512)	30.625 (3.472)	33.737 (3.672)	28.853 (2.615)	
	CAR1600.3	19.950 (0.735)	24.350 (1.075)	31.383 (4.331)	45.104 (3.698)	28.953 (5.021)	
	STO.18	32.225 (2.664)	38.025 (4.882)	31.750 (3.953)	22.476 (2.688)	41.471 (1.894)	


(continuation)


		Female	
		Abdijan 96	Tai30	cam115	OBAT1200.12	
 
 
 
Male
 
 
 
 	STO.10	15.733 (2.824)	43.800 (3.579)	31.242 (1.738)	16.925 (2.412)	
	STO.4	40.517 (7.376)	46.417 (0.102)	36.042 (8.515)	23.567 (2.817)	
	STO.7	26.583 (2.831)	24.900 (7.335)	36.750 (9.288)	44.000 (3.613)	
	san13	33.854 (3.310)	41.933 (5.974)	29.925 (4.134)	24.975 (5.515)	
	san1	34.601 (4.848)	40.125 (2.739)	32.683 (4.491)	30.975 (0.289)	
	CAR1555.1	22.583 (1.041)	27.625 (0.976)	34.350 (4.824)	48.825 (1.262)	
	CAR1600.1	41.342 (4.487)	24.600 (5.600)	36.233 (9.519)	36.900 (4.851)	
	CAR1600.3	33.942 (7.325)	46.925 (4.392)	34.300 (3.831)	40.442 (2.160)	
	STO.18	40.333 (5.307)	25.675 (5.467)	42.833 (4.362)	27.633 (5.212)	
