# Supplementary material for: Reinforcement of Gametic Isolation in Drosophila
Source: PLoS Biol. 2010 Mar 23;8(3):e1000341. doi: 10.1371/journal.pbio.1000341 (PMC2843595; doi:10.1371/journal.pbio.1000341)
Supplement: Table S7 — F1 ( D. yakuba × D. santomea ) larvae survival as a proxy for postzygotic isolation. Hybrid larvae from matings between females from allopatric or sympatric lines of D. yakuba and D. santomea were collected as first-instar larvae, and the number of recovered adults was scored. N equals 100 for all crosses. The data were analyzed with a nested ANOVA in which the asin (proportion of surviving larvae) was the response. The fixed effects were female line, nested within origin of the D. yakuba line (allopatric or sympatric) and male line nested within male origin. Female and male line effects caused heterogeneity (Female line: F 4,3589 = 65.39, p = 8.29×10^−16^; Male line: F 4,3589 = 53.011, p = 2.12×10^−15^), but there was no correlation between copulation latency and whether the populations were sympatric or allopatric (Female origin: F 1,3589 = 1.84, p = 0.1192; Male origin: F 1,3589 = 0.6155, p = 0.582). (0.03 MB RTF) [file pbio.1000341.s012.rtf]

Supplementary Table 7

	 	Female	
	 	BAR1000.2	OBAT1200.5	SA3	cam115	A2P	SJ2	
Male


	san13	68.420 (0.630)	56.980 (0.931)	71.920 (0.828)	58.243 (0.893)	62.270 (0.408)	67.059 (0.548)	
	STO.4	62.710 (0.380)	68.241 (0.807)	66.330 (0.849)	53.630 (0.740)	66.850 (1.096)	50.310 (1.051)	
	STO.18	55.980 (1.057)	60.195 (0.660)	61.744 (0.111)	57.153 (0.366)	54.186 (0.466)	56.333 (0.735)	
	CAR1566.6	62.544 (0.765)	55.219 (0.391)	74.080 (0.892)	55.404 (0.198)	59.740 (0.720)	54.333 (0.735)	
	CAR1600.1	64.520 (0.612)	51.940 (0.514)	50.320 (1.023)	58.490 (0.254)	62.594 (0.443)	55.140 (0.173)	
	CAR1600.3	57.368 (0.462)	62.320 (0.771)	54.169 (0.735)	54.205 (0.815)	57.419 (0.727)	60.392 (0.271)	
